# E2F4-Based Gene Therapy Mitigates the Phenotype of the Alzheimer’s Disease Mouse Model 5xFAD

**DOI:** 10.1007/s13311-021-01151-1

**Published:** 2021-11-11

**Authors:** Noelia López-Sánchez, Alberto Garrido-García, Morgan Ramón-Landreau, Vanesa Cano-Daganzo, José M. Frade

**Affiliations:** Department of Molecular, Cellular and Developmental Neurobiology, Cajal Institute, 28002 Madrid, Spain

**Keywords:** E2F4 phosphorylation, Aβ deposits, Neuroinflammation, Neuronal tetraploidy, Y-maze

## Abstract

After decades of unfruitful work, no effective therapies are available for Alzheimer’s disease (AD), likely due to its complex etiology that requires a multifactorial therapeutic approach. We have recently shown using transgenic mice that E2 factor 4 (E2F4), a transcription factor that regulates cell quiescence and tissue homeostasis, and controls gene networks affected in AD, represents a good candidate for a multifactorial targeting of AD. Here we show that the expression of a dominant negative form of human E2F4 (hE2F4DN), unable to become phosphorylated in a Thr-conserved motif known to modulate E2F4 activity, is an effective and safe AD multifactorial therapeutic agent. Neuronal expression of hE2F4DN in homozygous 5xFAD (h5xFAD) mice after systemic administration of an AAV.PHP.B-hSyn1.hE2F4DN vector reduced the production and accumulation of Aβ in the hippocampus, attenuated reactive astrocytosis and microgliosis, abolished neuronal tetraploidization, and prevented cognitive impairment evaluated by Y-maze and Morris water maze, without triggering side effects. This treatment also reversed other alterations observed in h5xFAD mice such as paw-clasping behavior and body weight loss. Our results indicate that E2F4DN-based gene therapy is a promising therapeutic approach against AD.

**Supplementary Information:**

The online version contains supplementary material available at 10.1007/s13311-021-01151-1.

## Introduction

Alzheimer’s disease (AD) is a progressive brain disorder that causes dementia in a substantial proportion of the elderly [1]. This condition is characterized by synaptic dysfunction and neuronal degeneration, accompanied by the alteration of spatiotemporal perception and other higher brain functions, including memory and language skills [2]. AD is also associated with extracellular deposits of β-amyloid peptide (Aβ) and neurofibrillary tangles composed of hyperphosphorylated tau protein [3], as well as physiological alterations including body weight loss [4, 5].

The etiology of AD is complex [6], and several pathological processes seem to take place at the early stages of the disease, including synapse loss [7], altered glucose metabolism [8], oxidative stress [9], chronic hypoperfusion [10], neuroinflammation [11], and neuronal cell cycle re-entry [12]. The latter is followed by full DNA replication that results in neuronal tetraploidy [13], synaptic dysfunction [14, 15], and the specific death of hyperploid neurons at later stages [14, 16, 17].

The complexity of the AD etiology may explain why, to date, no effective therapies are available for this disease, since the experimental therapies developed so far have mainly been focused on single targets [6] or a combination of specific drugs [18]. We have recently shown that the transcription factor E2 factor 4 (E2F4) could be a multifactorial target for AD [19]. Indeed, E2F4 is a major regulator of most AD-specific gene networks [20], whereas a number of other bioinformatics-based studies further indicate that E2F4 could participate in this disease [21–23]. Moreover, distinct AD-related genes contain binding sites of the E2F transcription factor family [24], and a genetics-based study has shown that a single nucleotide polymorphism that modifies a DNA binding motif of E2F4 is relevant for the disease [25]. In addition, biochemical evidence demonstrates that over 7000 genes involved in several processes that may underlie AD are potentially regulated by E2F4. This includes cell cycle gene regulators, as well as genes involved in DNA repair, RNA processing, stress response, apoptosis, ubiquitination, protein transport and targeting, protein folding, and I-κB kinase/NF-κB cascade [26]. Surprisingly, E2F4 could also play a direct role on synaptic function and memory formation since it can physically interact with relevant synaptic regulators including fragile X mental retardation 1 (FMR1), fragile X mental retardation syndrome-related protein 1 (FXR1), FXR2, and IQ motif and Sec7 domain-containing protein 2 (IQSEC2) [27], as well as with subunit 2 of biogenesis of lysosomal organelles complex-1 (BLOC-1), BLOC-1-related complex subunit 5, and SNARE-associated protein snapin [27], which are crucial for intracellular vesicle trafficking and synaptic vesicle recycling [28]. Other E2F4 interactors described by this latter study [27] are PCMT1/PIMT, an enzyme that repairs abnormal L-isoaspartyl linkages in age-damaged proteins [29], as well as two members of a molecular chaperone complex that plays an important role in proteostasis and contributes to the formation of nontoxic Aβ aggregates in vitro, prefoldin 1 (PFDN1) and PFDN4 [30]. E2F4 could therefore fulfill AD-relevant functions that are independent of its DNA binding activity.

E2F4 phosphorylation at its conserved Thr261/Thr263 motif in chicken (ortologous of Thr249/Thr251 in mouse and Thr248/Thr250 in human E2F4) can modulate its activity [31], thus resulting in an attractive therapeutic target. In this regard, we have shown that a dominant negative form of E2F4 (E2F4DN), containing Thr > Ala substitutions in this conserved Thr motif, blocks neuronal cell cycle re-entry [31] and neuronal tetraploidization (NT) [19, 32] and potentiates a transcriptional program consistent with global brain homeostasis, which attenuates neuroinflammation and modulates Aβ proteostasis [19]. This correlates with prevention of cognitive impairment and physiological alterations associated with AD such as body weight loss [19].

In this study, we have developed an AAV.PHP.B-based vector able to cross the blood–brain barrier in mice [33]. This allows to specifically express a human form of E2F4DN at physiological levels in neurons. This vector has been assayed in wild-type (WT) mice to confirm the absence of side effects of the E2F4DN-based gene therapy. This vector has also been administered to homozygous 5xFAD (h5xFAD) mice [34], resulting in the mitigation of the pathological phenotype observed in these mice.

## Materials and Methods

### Mice

Experimental procedures with mice were approved by the Consejo Superior de Investigaciones Científicas (CSIC) animal ethics committee and the Autonomous Government of Madrid, in compliance with the Spanish and European Union guidelines. Control C57BL/6JOlaHsd mice were purchased from ENVIGO (Barcelona, Spain). Double transgenic mice in C57BL/6 J genetic background expressing under the control of the Thy1 promoter both mutant human Aβ precursor protein 695 (APP_695_) with the Swedish (K670N, M671L), Florida (I716V), and London (V717I) FAD mutations and human presenilin 1 (PS1) harboring the M146L and L286V FAD mutations (Tg6799 or 5xFAD mice) [35] were purchased from The Jackson Laboratory (Bar Harbor, ME) (strain #008,730). 5xFAD mice were genotyped as indicated by The Jackson Laboratory. A h5xFAD mouse strain was obtained after repeated inbreeding of originally hemizygous mice. h5xFAD mice always yielded genetically homogeneous litters when crossed with WT mice (*n* = 20 litters). Homozygosity was confirmed by qPCR genotyping using specific primers of the human *APP* transgene (upstream primer: 5′-AGGATGGTGATGAGGTAGAG-3′ (from NM_201414 sequence) and downstream primer complementary to: 5′-CTGCTGTTGTAGGAACTCGA-3′ (896–915 bp and 1011–1030 bp from the NM_201414 sequence, respectively)), with the *Kcn3a* genomic sequence (see below) as a reference.

### Antibodies

The immunopurified rabbit anti phosphoThr249-E2F4 polyclonal antibody (pAb), generated with the support of the U2 facility (Custom Antibody Service, CAbS-IQAC-CSIC, Barcelona, Spain) of the singular scientific technical infrastructure “NANOBIOSIS” (http://www.nanbiosis.es/portfolio/u2-custom-antibody-service-cabs), was used at 1/500 dilution for western blot. This antibody was raised using a protocol based on affinity chromatography with phospho-[CSPQLT(pT)PTP] and dephospho-peptide [CSPQLTTPTP] columns [36]. The goat pAb to E2F4 (A85159; antibodies.com), identical to ab181483 (abcam, Cambridge, UK), was diluted 1/3000 for western blot. The mouse anti-Actin monoclonal antibody (mAb), clone C4 (MAB1501R; Merck Millipore, Burlington, MA) was used at 1/10,000 dilution for western blot. The rabbit anti-Myc tag pAb (ab9106; abcam) was used at 1/1000 for western blot and at 1/500 for immunohistochemistry. The rabbit anti-NeuN pAb (ABN78; Merck Millipore) was diluted 1/800 for flow cytometry. The mouse anti-NeuN mAb clone A60 (MAB377; Merck Millipore) was used at 1/1000 dilution for immunohistochemistry. The rabbit anti-GFAP pAb (ab7260; abcam) was diluted 1/1000 for immunohistochemistry. The mouse anti-GFAP mAb, Clone G-A-5 (G3893; Sigma-Aldrich, Burlington, MA), was 1/1000 diluted for immunohistochemistry. The rabbit anti-Iba1 pAb (019–19,741; FUJIFILM Wako Chemicals Europe GmbH, Neuss, Germany) was used at 1/800 dilution. The recombinant rabbit anti-Lamin B1 mAb (EPR8985(B)) (ab133741; abcam) was used at a 1/5000 dilution for western blot. The rabbit anti-β-Amyloid pAb #2454 (Cell Signaling Technology; Danvers, MA) was diluted 1/1000 for western blot. The mouse anti-APP A4 mAb, clone 22C11 (Invitrogen; Waltham, MA) was used at a dilution of 1/3000 for western blot.

The donkey anti-rabbit IgG (H + L) highly cross-adsorbed secondary antibody; Alexa Fluor 488 (Invitrogen) was used at 1/1000 dilution for immunohistochemistry and 1/400 for flow cytometry. The goat anti-mouse IgG (H + L) cross-adsorbed secondary antibody; Alexa Fluor 568 (Invitrogen) was diluted 1/1000 for immunohistochemistry. The IRDye 800CW Goat anti-Rabbit IgG (H + L), IRDye 680RD Goat anti-Mouse IgG (H + L), and IRDye 800CW Donkey anti-Goat antibodies (LI-COR, Lincoln, NE) were diluted 1/15,000 for western blot.

### Recombinant E2F4DN Protein

A recombinant human E2F4DN protein, StrepII-tagged at its C-terminus (hE2F4DN-StrepII), was produced by Advanced Biotech (Madrid, Spain) using baculovirus-infected Sf21 insect cells grown in suspension in animal-derived component-free (ADCF) medium (HyClone SFM4Insect) (yield, 0.64–0.75 mg per liter of culture). Transfected cells were cultured for 84 h and then harvested by centrifugation. Cell pellets were resuspended in Hep-A buffer (20 mM sodium potassium phosphate, 0.1 mM EDTA, 2 mM dithiothreitol, 5% v/v glycerol, pH 7.0) and lysed by sonication. DNA was eliminated by incubating the crude lysate with Pierce Universal Nuclease (Thermo Fisher Scientific, Waltham, MA) for 20 min at 4 °C, and free biotin and biotinylated proteins were blocked by adding 1 U avidin (IBA Lifesciences, Göttingen, Germany) to the crude lysate. Finally, the cell debris and insoluble matter were removed by centrifugation. The supernatant was then loaded onto a StrepTrap HP 1 ml column (GE Healthcare, Wauwatosa, WI) mounted on an ÅKTA Pure 25 protein purification system (Cytiva; Marlborough, MA), washed with 20 column volumes of Hep-A buffer, and then eluted with Hep-A buffer supplemented with 50 mM desthiobiotin (IBA Lifesciences). Fractions containing the purified hE2F4DN-StrepII were pooled together, flash frozen, and stored at −80 °C. Finally, the identity of the purified protein was confirmed with E2F4-specific antibodies.

### AAV.PHP.B Vectors

The human E2F4 coding sequence with Thr248Ala/Thr250Ala mutations and containing the Kozak sequence (ACCATGG) and either a final stop codon (hE2F4DN) or the *c-Myc* tag sequence plus a stop codon (hE2F4DN-myc) was synthesized (GeneScript, Piscataway, NJ) and cloned into the pcDNA 3.1( +) vector (Invitrogen). After confirming the sequences, they were PCR amplified and seamless cloned [37] into the KD10 plasmid (Packgene, Worcester, MA), flanked by the human synapsin 1 (*hSyn1*) promoter [38] and the mutant woodchuck hepatitis virus post-transcriptional regulatory element (WPRE) sequence described by [39] (WPRE3SL), which lacks tumorigenic capacity [39]. The WPRE3SL cassette has the SV40 late polyadenylation signal sequence plus the upstream polyadenylation enhancer element repeated in tandem described by Schambach et al. [40]. The expected sequence was confirmed by Sanger sequencing. Research-grade, single-stranded AAV.PHP.B.hSyn1.hE2F4DN-myc.WPRE3SL (AAV-hE2F4DN-myc), AAV.PHP.B.hSyn1.hE2F4DN.WPRE3SL (AAV-hE2F4DN), and AAV.PHP.B.hSyn1.EGFP.WPRE3SL (AAV-EGFP) vectors for preclinical testing were manufactured by Packgene by co-transfecting the respective KD10 plasmids together with the pAdDeltaF6 helper vector containing essential adenoviral elements (E4 transcription unit, viral-associated RNA, and adenoviral DNA-binding protein) and the pRep2-Cap-PHP.B vector expressing both Rep- and PHP.B-specific Cap proteins [33]. The final purified product was dialyzed in phosphate-buffered saline (PBS) with additional 0.001% Pluronic F-68. Viral titer was determined by qPCR and confirmed by silver stain. AAV.PHP.B vectors were intravenously (i.v.) administered through the tail vein at 3.12 × 10^13^ vg/kg, 6.25 × 10^13^ vg/kg, or 1.25 × 10^14^ vg/kg. See Table S1 for a comprehensive list of experiments performed with these vectors.

### Genomic DNA Extraction

Genomic DNA was extracted after overnight (O/N) incubation of the mouse tail tips with 1 ml lysis buffer (100 mM Tris–HCl pH 8.5, 5 mM EDTA, 0.2% SDS, 200 mM NaCl, 0.1 mg/ml proteinase K) at 55 °C, followed by addition of 440 µl de 5 M NaCl and incubation on ice for 10 min. Extracts were then centrifuged at 16,000 × *g* for 15 min at 4 °C, and cold ethanol was added to the supernatants to reach final concentration of 65%. DNA was pelleted by centrifugation as above and washed twice with cold 70% ethanol followed by an additional centrifugation step. Pellets are then dried for 5–10 min at 55 °C and resuspended in water. Samples were finally maintained at 55 °C and subjected to several cycles of sonication (10 min each) to eliminate viscosity.

### RNA Extraction and cDNA Synthesis

Total RNA from tissues of mice treated with AAV.E2F4DN-myc or AAV.E2F4DN was extracted using QIAzol Reagent (Qiagen, Hilden, Germany), and cDNA was synthesized using SuperScript IV Reverse Transcriptase (Thermo Fisher Scientific) following the indications of the manufacturer.

### qPCR

**Fig. 1:**
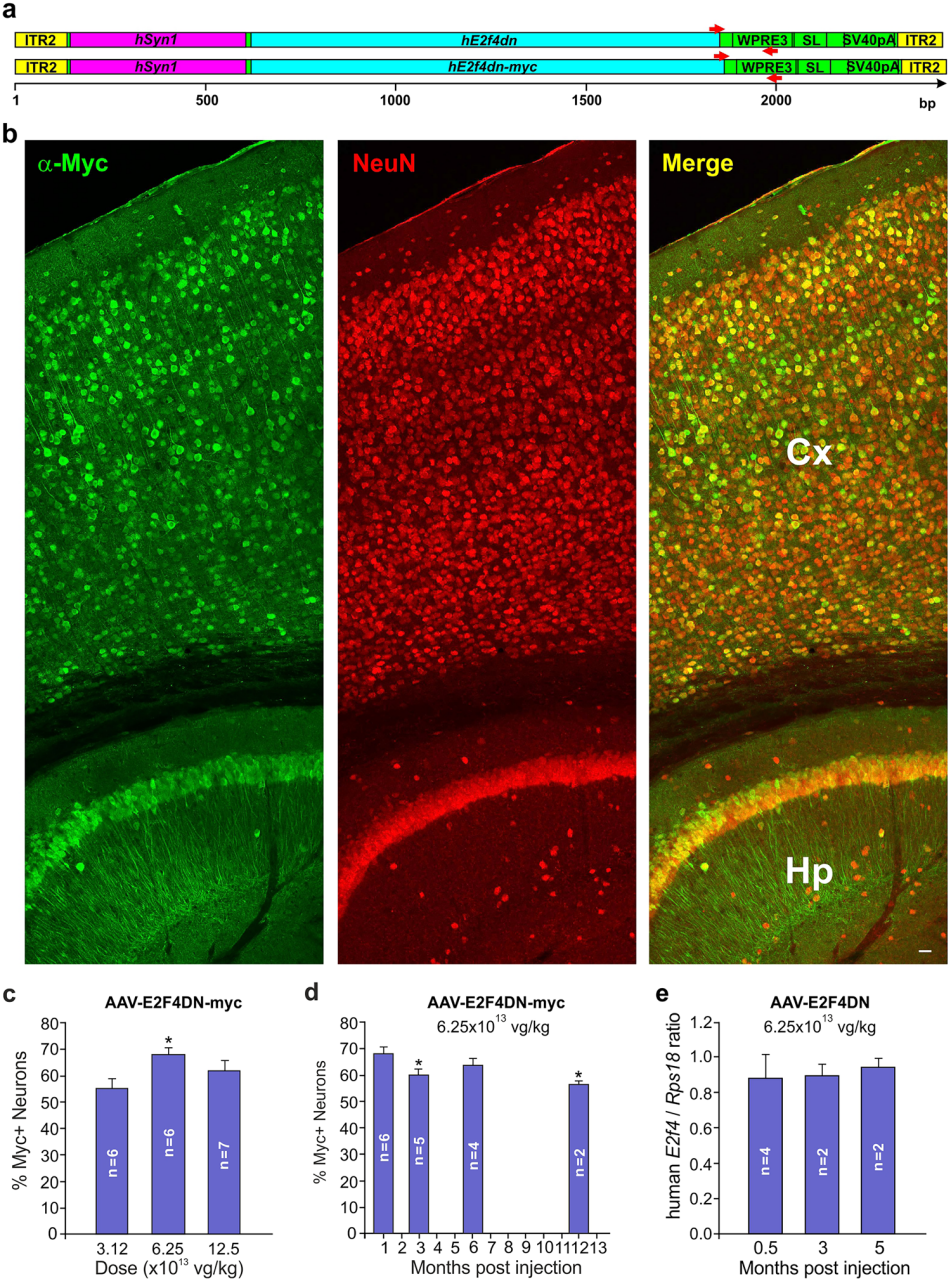
Expression of E2F4DN-myc in the cerebral cortex of WT mice subjected to systemic administration of the AAV-hE2F4DN-myc vector at 6 weeks of age. (**a**) Scheme showing the genomic sequences of AAV-hE2F4 (top) and AAV-hE2F4-myc (bottom) vectors. Yellow: ITR sequences from AAV2. Pink: *hSyn1* promoter. Blue: *E2f4dn* sequence without (top) or with (bottom) a myc tag. Green: WPRE3SL sequence including the SV40 polyadenylation signal. Small red arrowheads: primers used for qPCR and qRT-PCR. (**b**) A vibratome section of the cerebral cortex (Cx) and hippocampus (Hp) from WT mice injected at 1.5 months of age with the AAV-hE2F4DN-myc vector (6.25 × 10^13^ vg/kg) and sacrificed 1 month later, immunostained with anti-Myc tag (green) and NeuN (red). (**c**) Percentage of cortical neurons expressing E2F4DN-myc 1 month after delivery (at 1.5 months) of the AAV.E2F4DN-myc vector at the indicated doses. (**d**) Percentage of cortical neurons expressing E2F4DN-myc at the indicated time points after delivery (at 1.5 months) of the AAV.E2F4DN-myc vector at 6.25 × 10^13^ vg/kg. (**e**) Ratio between *hE2f4dn* and *Rps18* gene expression, quantified by qRT-PCR from cDNA obtained from the cerebral cortex of h5xFAD mice injected at 1.5 months of age with the AAV-hE2F4DN vector (6.25 × 10^13^ vg/kg) and sacrificed at the indicated time points. *p < 0.05 (*F* = 10.8002; one-way ANOVA plus Tukey’s HSD post hoc test). Scale bar, 10 μm

qPCR was performed with either the SsoAdvanced Universal SYBR Green Supermix (Bio-Rad, Hercules, CA) (for transgene quantification) or the qPCRBIO SyGreen Mix (PCRBIOSYSTEMS, London, UK) (genotyping), using a 7500 Real-Time PCR equipment (Applied Biosystems, Waltham, MA). A fragment of the mouse *Kcn3a* gene was amplified using an upstream primer with sequence 5′-TCCAACTTCAACTACTTCTACCAC-3′ and a downstream primer with sequence 5′-CACCATATACTCCGACTTACTCAG-3′, located respectively at base pairs (bp) 1561–1584 and 1690–1713 of the *Kcn3a* sequence (NM_008418). A fragment of the mouse *Rps18*-specific cDNA was amplified by qRT-PCR as above using an upstream primer with sequence 5′-AATAGCCTTCGCCATCACTG-3′ and a downstream primer with sequence 5′-GTCTGCTTTCCTCAACACCA-3′ located respectively at 120–139 and 173–192 bp of the *Rps18* cDNA (NM_011296). Both *E2F4dn* mRNA and the AAV-E2F4DN and AAV-E2F4DN-myc vector genomic DNAs were amplified as above with an upstream primer with sequence 5′-TGATGTGCCTGTTCTCAACC-3′ and a downstream primer with sequence 5′-GCATTAAAGCAGCGTATCCAC-3′, which localize to the vector sequence illustrated in Fig. 1a (red arrowheads). The ratio AAV-E2F4DN genomic DNA/*Kcn3a* was multiplied by 2 to estimate the amount of viral genomes per diploid genome.

### Thioflavin S Staining

Vibratome sections were washed three times with phosphate-buffered saline (PBS) containing 0.4% Triton X-100 (Sigma-Aldrich) (0.4% PBTx) and then incubated for 30 min in the dark with 0.05% thioflavin S (Sigma-Aldrich) in 50% ethanol (Merck). Finally, sections were washed twice with 50% ethanol and once with distilled water. Then, sections were subjected to immunohistochemistry as described below.

### Immunohistochemistry

Mice were transcardially perfused with PBS, and then with 4% paraformaldehyde (PFA), and the brain, heart, kidney, adrenal gland, ovary, spleen, and liver were postfixed O/N at 4 °C with 4% PFA. Cryosections (15 μm) were permeabilized and blocked for 1 h at RT in phosphate-buffered saline (PBS)/0.1% Triton X-100 (Sigma-Aldrich) (0.1% PBTx) and 10% fetal calf serum (FCS; Invitrogen) and incubated O/N at 4 °C with the primary antibodies in 0.1% PBTx plus 1% FCS. After washing with 0.1% PBTx, the sections were incubated for 1 h at RT in 0.1% PBTx plus 1% FCS with the secondary antibodies. The sections were washed in 0.1% PBTx, and then, they were incubated with 100 ng/ml 4′,6-diamidine-2′-phenylindole dihydrochloride (DAPI; Sigma-Aldrich) in PBS before mounting with PBS/glycerol (1:1). For immunolabeling with anti-phosphoepitopes, Tris-buffered saline (TBS) was used instead of PBS. Vibratome sections (50 μm) were permeabilized and blocked in 0.4% PBTx containing 10% FCS for 4 h. Then, they were incubated O/N at 4 °C with the primary antibodies in 0.4% PBTx. After four washes of 20 min with 0.1% PBTx, sections were incubated with the secondary antibodies plus 100 ng/ml DAPI in 0.4% PBTx for 4 h at room temperature (RT). Sections were then washed four times with 0.1% PBTx and mounted with PBS/glycerol (1:1).

### Quenching of Lipofuscin Autofluorescence Signal

Lipofuscin was quenched with TrueBlack Lipofuscin Autofluorescence Quencher (Biotium, Fremont, CA). Briefly, vibratome sections were washed once with PBS and treated for 30 s with TrueBlack 1 × prepared in 70% ethanol. Finally, sections were washed three times with PBS and then immunostained as described above.

### Confocal Microscopy and Image Analysis

Confocal images were acquired at × 20 magnification with a Leica SP5 confocal microscope. Image analysis was performed using ImageJ (Fiji). Images used for the analysis (at least two mosaic images per tissue and animal) were maximum intensity projections, created as output images whose pixels correspond to the maximum value of each pixel position (in *xy*) across all stack images (*z*). For the analysis of the area occupied by Iba1 and GFAP, threshold was determined to highlight the area to be quantified. If necessary, the region of interest (ROI) tool was then used to delimitate the different cortical layers. For the analysis of the area occupied by thioflavin S, the filter mean was applied with value 2; then, a threshold was determined to highlight the area to be quantified and the plugin Analyze Particles was then used. Counting of Myc-positive/NeuN-positive nuclei in brain sections was performed using z-projections since NeuN labeling and Myc labeling occasionally localize in different subcellular compartments (nucleus *vs* cytoplasm).

### Western Blot

Extracts from cerebral cortex were obtained in cold extraction buffer (20 mM Tris–HCl pH 6.8, 10 mM β-mercaptoethanol, 1 mM EDTA, 1% Triton X-100, 1% SDS) including 1 × cOmplete Mini, EDTA-free, protease inhibitor cocktail (Roche, Basel, Switzerland) (one hemicortex in 500 μl extraction buffer). Extracts were centrifuged for 10 min at 14,000 × *g* (at 4 °C), and supernatants were then boiled for 5 min in either loading buffer tricine (50 mM Tris–HCl pH 8.0; 12% glycerol, 4% SDS, 0.01% Coomassie blue, 2% β-mercaptoethanol), for Aβ analysis, or Laemli buffer, for other analyses. Extracts were fractionated by SDS PAGE on 16.5% Tris-tricine acrylamide gels (Bio-Rad) and transferred to either 0.2-μm low-fluorescence PVDF transfer membranes (Thermo), for  Aβ analysis, or SDS PAGE on 11%μ Tris-glycine acrylamide gels and transferred to 0.45 μm Immobilon-FL transfer membranes (Millipore), for other analyses. The membranes were incubated for 1 h with Odyssey blocking buffer (LI-COR) in TBS (OBB) and then incubated ON at 4 °C with the appropriate antibody in OBB containing 0.1% Tween 20. After washing the membranes five times in TBS containing 0.1% Tween 20 (TBS-T), they were incubated for 1 h at RT with a 1/15,000 dilution of secondary antibodies in OBB containing 0.1% Tween 20. Finally, they were washed again with TBS-T as described above, and the protein bands were visualized using the Odyssey CLx Infrared Imaging System (LI-COR).

### Cell Nuclei Isolation

Cell nuclei isolation was performed as described by López-Sánchez and Frade [41]. Briefly, fresh-frozen mouse cerebral hemicortices were placed in 2.5 ml ice-cold, DNase-free 0.1% PBTx and protease inhibitor cocktail (Roche) (nuclear isolation buffer). Cell nuclei were then isolated by mechanical disaggregation using a dounce homogenizer. Undissociated tissue was removed by centrifugation at 200 × *g* for 1.5 min at 4 °C. The supernatant was eightfold diluted with nuclear isolation buffer and centrifuged at 400 × *g* for 4 min at 4 °C. Supernatant with cellular debris was discarded, and the pellet incubated at 4 °C in 800–1000 μl cold nuclear isolation buffer for at least 1 h, prior to mechanical disaggregation by gently swirl of the vial. The quality and purity of the isolated nuclei were analyzed microscopically after staining with 100 ng/ml DAPI.

### Flow Cytometry

Flow cytometry was carried out as described by López-Sánchez and Frade [41]. Immunostaining of cell nuclei was performed by adding both primary (rabbit anti-NeuN) and secondary (Alexa 448-coupled anti-Rabbit) to 400 μl of isolated unfixed nuclei containing 5% of fetal calf serum (FCS) and 1.25 mg/ml of BSA. In control samples, the primary antibody was excluded. Finally, the reaction was incubated O/N at 4 °C in the dark. Immunostained nuclei (400 μl) were filtered through a 40-μm nylon filter, and the volume adjusted to 800–1000 μl with DNase-free 0.1% PBTx containing propidium iodide (PI; Sigma-Aldrich) and DNAse-free RNAse I (Sigma-Aldrich) at a final concentration of 40 μg/ml and 25 μg/ml, respectively, and incubated for 30 min at RT. The quality of the nuclei and specificity of immunostaining signal was checked by fluorescence microscopy. Flow cytometry was then carried out with a FACSAria I cytometer (BD Biosciences, San Diego, CA) equipped with a 488-nm Coherent Sapphire solid state and 633-nm JDS Uniphase HeNe air-cooled laser. Data were collected by using a linear digital signal process. The emission filters used were BP 530/30 for Alexa 488 and BP 616/23 for PI. Data were analyzed with FACSDiva (BD Biosciences) and Weasel 3.0.1 (Walter and Eliza Hall Institute of Medical Research) software and displayed using biexponential scaling. Electronic compensation for fluorochrome spectral overlap during multi-color immunofluorescence analysis was carried out when needed. Cellular debris, which was clearly differentiated from nuclei due to its inability to incorporate PI, was gated and excluded from the analysis. DNA content histograms were generated excluding doublets and clumps by gating on the DNA pulse area *versus* its corresponding pulse height. The exclusion of doublets was confirmed by checking the DNA pulse area *versus* the pulse width of the selected population, and the percentage of tetraploid nuclei was quantified. A minimum of 15,000 and 20,000 nuclei were analyzed for the NeuN-positive population. The proportion of tetraploid nuclei was normalized to the value obtained in cell nuclei from control 2-month-old WT mice as shown by López-Sánchez et al. [42], which was used as internal control in all the experiments.

### Morris Water Maze (MWM)

The MWM test [43] was used to evaluate spatial learning. The apparatus was a circular tank 100 cm in diameter, full of water (at 21–22 °C). Inside the tank, a transparent platform was hidden in the same location 2 cm below the water level. The experiment consisted of four sessions including four trials 30–60 min apart performed under constant illumination conditions (7–15 lx). Each of the four starting positions (N, S, E, W) was used randomly in every daily session. Each trial was terminated when the mouse located the platform or when 60 s had elapsed; there then followed a period of 15–20 s, in which the animal was allowed to stay on the platform. Several fixed extra maze cues were constantly visible from the pool. All trials were videotaped by a camera located 2 m above the water level. Mice trajectories were analyzed using the Ethovision 3.1 computerized tracking system (Noldus, Wageningen, The Netherlands), to measure escape latency for each animal in each trial, and the average latency was calculated for each day. The average velocity for each animal throughout all the trials was also calculated.

### Spontaneous Alternation Y-Maze Test

Each mouse was placed into the center of a Y-maze apparatus (Panlab) and then allowed to freely explore the different arms during an 8-min session [44]. The sequence of arms entered was recorded, and working memory was measured as the percentage of alternation (p.a.), which was calculated as the number of triads containing entries in all three arms divided by all the triads and then multiplied by 100.

### Elevated Plus Maze Test (EPM)

Each mouse was subjected to a single 5-min trial in an EPM apparatus for mouse (med associates, Inc.), which consisted of two closed arms (5 cm wide × 30 cm long, with clear perplex walls 15 cm high), and two open arms (5 cm × 30 cm) raised 40 cm from the floor. During each trial, the mice were placed in the center of the maze and allowed to move freely along the apparatus under a constant intense white light. The movement of the animals was recorded and analyzed using Ethovision software (Noldus), and the data are presented as the total time (s) spent standing or walking in the closed arms (an indication of anxiety), in the open arms, or in the hub.

### Clasping Test

To test clasping behavior, mice were suspended by the tail and recorded for 15 s. A score was given on a scale from 0 to 3 as described by Jawhar et al. [45] (0 = no clasping, 1 = forepaws clasping, 2 = forepaws and one hind paw clasping, and 3 = all paws clasping). Two investigators independently evaluated and scored the behavior in a blinded manner. Equivalent results were obtained for both evaluations.

### Cell Counting

For quantification of the proportion of cortical neurons expressing hE2F4DN-myc, confocal images including all cortical layers were obtained from previously immunostained vibratome sections. Confocal projection images (16–20 μm thickness) from both motor and sensory cortex were evaluated. At least, three images per each cortical region were analyzed per mouse. Above 10,000 NeuN-positive nuclei were counted per mouse. Numbers from both cortical regions were combined to get the final quantification.

### Statistical Analyses

Measurements were taken for distinct samples, which for the study of temporal progression of body weight, percentage of survival, and training learning in the MWM paradigm were measured repeatedly. Quantitative data are represented as the mean ± SEM. Statistical differences in experiments performed with transgenic mice on either EGFP or E2F4DN background were analyzed using two-tailed Student’s *t* test. One-way ANOVA analysis was performed in AAV-based experiments, followed by Tukey’s honestly significant difference (HSD) post hoc test. Two-way ANOVA analysis was performed in AAV-based experiments, followed by Student’s *t* post hoc test. Outliers, as evidenced by the Grubbs’ test, were eliminated from the analysis. The Kruskal–Wallis test was used for analysis of the paw-clasping test and of the EPM test [time in open arms/(time in open + closed arms)] in as these variables are not normally distributed. Statistical significance of the Pearson’s *r* correlation value was obtained using the SPSS Statistics package (IBM). Statistical significance of differences between two linear regression slopes was evaluated using a *t* test based on each line's slope, standard error, and sample size (https://www.real-statistics.com/regression/hypothesis-testing-significance-regression-line-slope/comparing-slopes-two-independent-samples/). Median survival of mice after treatment (in weeks) was evaluated as the length of time from the start of treatment that half of the mice included a defined experimental group are still alive.

## Results

### Systemic Delivery Through an AAV.PHP.B Vector Allows the Expression of hE2F4DN-myc in Cortical Neurons

Based on previous genetic proof of concept based on a transgenic mouse strain showing neuron-specific expression of a Thr249Ala/Thr251Ala mutant form of mouse E2F4, *Myc* tagged at its C-terminus (E2F4DN-myc) [19], we hypothesized that human E2F4DN (hE2F4DN), containing Ala mutations in the Thr248/Thr250 motif, could be used as a therapy for AD. To explore methods for delivery of human E2F4DN (hE2F4DN) to the Alzheimer brain, we focused on the adeno-associated vector AAV.PHP.B, a variant of AAV9 with enhanced capacity to cross the blood–brain barrier (BBB) in mice [33]. An AAV.PHP.B vector was therefore generated, carrying hE2F4DN, *Myc* tagged at the C-terminus, under the control of the neuron-specific, *hSyn1* promoter [38], followed by a modified version of the WPRE that improves transcript processing [39, 46, 47] and expression in neurons [48], as well an enhancement of *hSyn1* promoter strength [49], modified to prevent tumorigenic capacity [38] (Fig. 1a). Hereafter, we refer to this vector as AAV-hE2F4DN-myc.

Different doses of AAV-hE2F4DN-myc (3.12, 6.25, and 12.50 × 10^13^ vg/kg) were intravenously injected in WT mice of 1.5 months of age to verify its capacity to efficiently deliver hE2F4DN-myc to the brain. This analysis demonstrated that hE2F4DN-myc was observed in neurons (NeuN-positive cells) (Fig. 1b) but not in astrocytes (Fig. S1), a cell type known to be strongly transduced by AAV.PHP.B [50]. A quantitative analysis indicated that around ~70% of NeuN-positive neurons expressed detectable levels of hE2F4DN-myc 1 month after systemic delivery of the AAV-hE2F4DN-myc vector at 6.25 × 10^13^ vg/kg, the lower dose at which maximal transduction was detected (Fig. 1c). Therefore, this dose was employed hereafter, independently of the transgene used. Using this dose, we confirmed that the AAV-hE2F4DN-myc vector can efficiently cross the BBB in older mice (8.5 months) since 50.77 ± 0.79% (*n* = 2) of neurons showed intense E2F4DN-myc immunostaining in cortical neurons 1 month postadministration (Fig. S2).

In our hands, the *hSyn1* promoter was able to induce long-lasting neuronal expression of hE2F4DN-myc in WT mice since the transgene was detected in ~55% of neurons 1 year after the administration of the AAV-hE2F4DN-myc vector (Fig. 1d).

Quantitative reverse transcription PCR (qRT-PCR), performed in the cerebral cortex of WT mice administered with the AAV-hE2F4DN-myc vector at 1.5 months of age and sacrificed 3 months later, indicated that *E2f4dn-myc* mRNA can be detected at comparable levels to those of control *Rps18* mRNA (human *E2F4*/*Rps18* ratio, 1.15 ± 0.19; *n* = 2). Therefore, this analysis confirmed the observation by Jackson et al. [51] that the *hSyn1* promoter can be used for neuronal expression at physiological levels of transgenes carried by the AAV.PHP.B vector.

### hE2F4DN-myc Expression in Cortical Neurons from WT Mice Does Not Trigger Adverse Effects

**Fig. 2:**
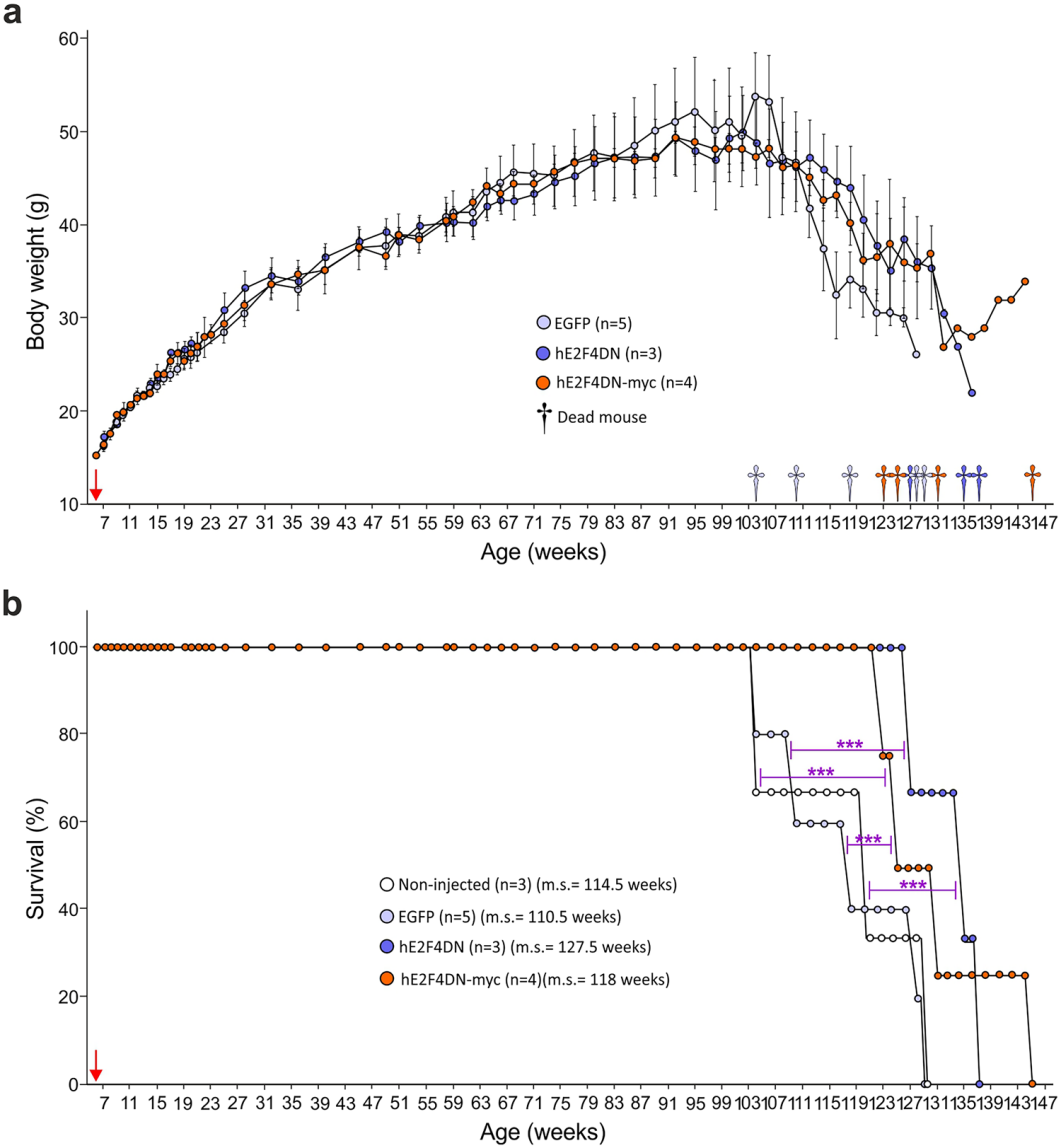
Systemic administration of the AAV-hE2F4DN/AAV-hE2F4DN-myc vectors does not affect body weight gain and increases life expectancy in female WT mice. (**a**) Body weight at the indicated time points of WT mice administered at 6 weeks with either AAV-EGFP (EGFP), AAV-E2F4DN (hE2F4DN), or AAV-hE2F4DN-myc (hE2F4DN-myc). Nonsignificant; two-tailed Student’s *t* test. (**b**) Survival expectancy in WT mice either noninjected or administered with AAV-EGFP, AAV-E2F4DN, or AAV-hE2F4DN-myc vectors. ***p < 0.001 (Non-injected *vs* E2F4DN: χ_(1)_ = 49.09;  Non-injected *vs* E2F4DN-myc: χ_(1)_ = 18.55; EGFP *vs* E2F4DN: *χ*_(1)_ = 32.41; EGFP *vs* E2F4DN-myc: *χ*_(1)_ = 32.41; log-rank (Mantel-Cox) test). Red arrow: time point at which the AAVs were administered. m.s. = median survival

As in our previous study using transgenic mice that express mouse E2F4DN-myc in neurons [19], AAV-hE2F4DN-myc vector administration did not trigger detectable adverse effects. Similar body weight gain was observed during the whole life span in WT mice subjected to systemic delivery of AAV-hE2F4DN-myc when compared with WT mice treated with a control AAV.PHP.B vector expressing EGFP under the control of the *hSyn1* promoter control (AAV-EGFP) (Fig. 2a). Furthermore, AAV-hE2F4DN-myc administration induced a statistically significant increase of life expectancy in mice when compared with AAV-EGFP-injected mice (Fig. 2b).

We also studied the biodistribution of hE2F4DN-myc protein by immunohistochemistry. We could not detect hE2F4DN-myc labeling in any of the analyzed organs (heart, spleen, ovary, kidney, adrenal gland, thymus, muscle, intestine, and lung) (Tables S1 and S2), except, marginally, in the heart. In this organ, only one cardiomyocyte was detected in one out of 184 cryosections (Tables S1 and S2). In the liver, a minor number of hepatocytes were observed to express hE2F4DN-myc 1 month after systemic administration of the AAV-hE2F4DN-myc vector (Tables S2 and S3). This number was reduced to one-third when analyzed by 3 or 6 months after delivery of the AAV-hE2F4DN-myc vector (Tables S2 and S3; Fig. S3a). Importantly, we did not detect E2F4DN-myc-positive cell clones in the liver at any time point, indicating that hE2F4DN-myc expression does not lead to tumor transformation. Finally, some Myc-positive cells were detected within the connective tissue surrounding the ovary 1 month postadministration (Fig. S3b), but not at later stages. We conclude that the *hSyn1* promoter is highly efficient to prevent transgene expression in cells other than neurons.

To rule out that AAV-based transduction of the hE2F4DN-myc transgene can result in long-lasting neurological alterations, we focused on paw-clasping behavior and anxiety. Paw-clasping is an abnormal reflex observed in several mouse strains with lesions in cerebellum, basal ganglia, and neocortex, as well as transgenic models of AD, characterized by the linking of their paws together, whereas suspended in the air [52]. The degree of paw-clasping was studied in 16-month-old WT mice administered at 1.5 months with either AAV-hE2F4DN or AAV-EGFP or left noninjected. This analysis indicated that E2F4DN administration does not enhance the clasping phenotype already observed in noninjected, WT mice at the analyzed age (Fig. S4). We also studied anxiety using the EPM paradigm [53] in 12-month-old WT mice administered at 1.5 months with either AAV-hE2F4DN-myc or control AAV-EGFP vectors or left noninjected. No statistical significant differences were observed between groups (Fig. S5). We therefore conclude that AAV-based transduction of the hE2F4DN-myc transgene does not result in evident neurological deficits.

Finally, long-term survival and body weight gain was analyzed in WT mice administered at 1.5 months with either AAV-hE2F4DN (see below), AAV-hE2F4DN-myc, or control AAV-EGFP vector. This analysis indicated that no significant variation in these parameters was observed during the first 2 years of treatment (Fig. 2a, b). Nevertheless, neuronal expression of hE2F4DN led to a tendency to reduced weight loss in mice aged above 2 years old whereas a statistically significant increase of life expectancy was observed in AAV-hE2F4DN and AAV-E2F4DN-myc-treated mice compared with AAV-EGFP-treated mice (Fig. 2a, b). Previous studies showed that GFP transduction can cause cellular damage [54]. Therefore, we decided to compare the survival of AAV-hE2F4DN and AAV-E2F4DN-myc treated mice with that of noninjected WT mice. This analysis confirmed that the expression of hE2F4DN, and of hE2F4DN-myc, results in longer life expectancy (Fig. 2b) (respective median survivals in weeks for noninjected and EGFP, hE2F4DN, and hE2F4DN-myc, 114.5, 110.5, 127.5, 118). In sum, neuronal expression of E2F4DN not only lacks side effects but also benefits life expectancy in WT mice.

### Both E2F4DN Viral Genome and *E2f4dn* mRNA Accumulates in the Cerebral Cortex of WT Mice Administered with an AAV.PHP.B-hE2F4DN Vector

To verify that E2F4DN can be efficiently delivered into the brain of WT mice, a new AAV.PHP.B vector was generated expressing hE2F4DN under the control of the *hSyn1* promoter and also harboring the modified WPRE sequence described above. This vector (hereafter referred to as AAV-hE2F4DN) was intravenously administered to WT mice of 1.5 months, and this treatment resulted in a similar profile of body weight gain and survival as that obtained with the AAV-E2F4DN-myc vector (Fig. 2), confirming that AAV-hE2F4DN lacks significant side effects.

The presence of the viral genome, normalized to an endogenous gene (*Kcn3a*), was evaluated by quantitative PCR (qPCR) in genomic DNA from the cerebral hemispheres 1 month postadministration. This analysis indicated that 3.86 ± 0.80 viral genomes per diploid genome can be detected in this tissue (Fig. S6a). The presence of the viral genome was also elevated in other neural derived structures (spinal cord and adrenal gland) as well as in tissues where a minority of E2F4DN-myc cells was observed (liver, heart and ovary; see above) (Fig. S6a). In addition, the AAV-hE2F4DN vector showed relatively high tropism for the lung, skeletal muscle, and spleen, whereas it showed low tropism for other tissues including intestine, kidney, and thymus (Fig. S6a).

To study whether AAV-E2F4DN transduction can result in *hE2F4dn* expression in all the above mentioned tissues, AAV-hE2F4DN was intravenously administered to 1.5-month-old, WT mice, and the presence of *hE2f4dn* mRNA, normalized to endogenous *Rps18* mRNA, was assessed by qRT-PCR in their cerebral hemispheres 3 months postadministration. We found that the cerebral cortex expressed *hE2f4dn* mRNA at similar levels as those of the house-keeping gene *Rps18* whereas the spinal cord expressed it at slightly higher levels than those of this latter gene (Fig. S6b). In contrast, the adrenal gland, liver, heart, ovary, lung, muscle, spleen, intestine, and thymus expressed *hE2f4dn* mRNA at quite low levels, whereas *hE2f4dn* mRNA was virtually undetectable in the kidney (Fig. S6b). Therefore, as with AAV-hE2F4DN-myc, AAV-based transduction of the hE2F4DN transgene seems to be highly specific of the nervous system.

### Systemic Delivery of hE2F4DN Using the AAV.PHP.B Vector Can Be Performed in h5xFAD Mice

Once we demonstrated that neither the AAV-hE2F4DN-myc nor the AAV-hE2F4DN vector triggers visible adverse effects on WT mice, we decided to test the capacity of the AAV.PHP.B vector to deliver therapeutic levels of hE2F4DN. Therefore, the AAV-hE2F4DN vector was intravenously administered into h5xFAD mice of 1.5 months of age. We decided to perform this analysis in h5xFAD mice because homozygosity results in an aggravated neurological phenotype that is visible already at 2 months [34]. At this age, h5xFAD mice show soluble Aβ_1–40_ in the brain (which is not yet detected in hemizygous mice), as well as a significant increase in Aβ plaques and reduced spatial memory [34].

We first studied whether AAV-E2F4DN transduction results in *hE2F4dn* expression in the central nervous system. Therefore, AAV-hE2F4DN was intravenously administered to 1.5-month-old, h5xFAD mice, and the presence of *hE2f4dn* mRNA, normalized to endogenous *Rps18* mRNA, was assessed by qRT-PCR in their cerebral hemispheres 2 weeks, 3 months, and 5 months postadministration. This analysis indicated that the expression of *E2f4dn* mRNA was maintained with time, showing similar levels as those of the endogenous *Rps18* mRNA (*hE2f4*/*Rps18* ratio ~0.9) (Fig. 1e).

### Systemic Delivery of hE2F4DN Using the AAV.PHP.B Vector Reduces Aβ Levels and Plaques in h5xFAD Mice

**Fig. 3:**
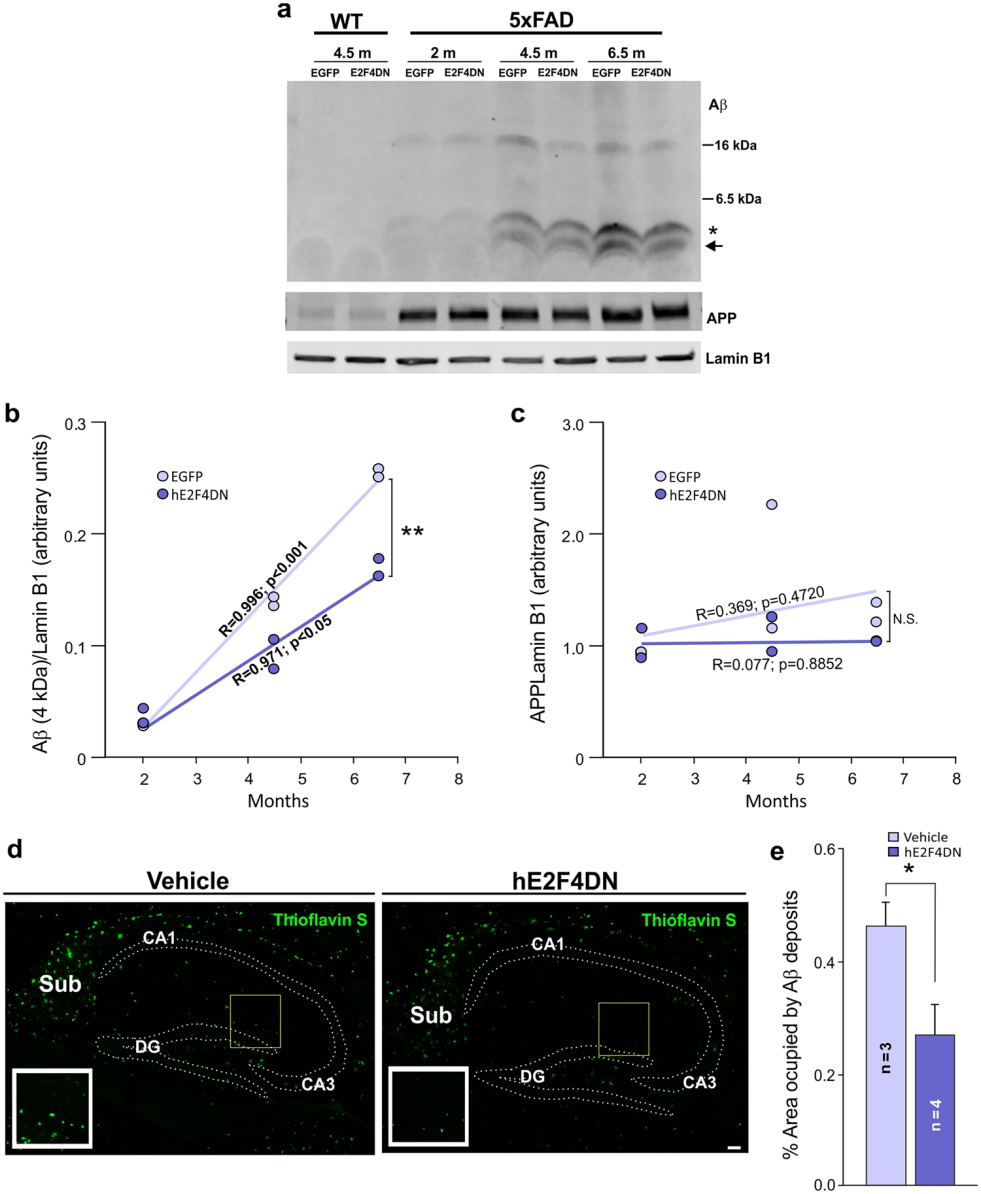
Systemic administration of the AAV-hE2F4DN vector reduces Aβ production and accumulation in the hippocampus in h5xFAD mice. (**a**) Representative western blot of hippocampal extracts from wild-type (WT) and h5xFAD mice of 2, 4.5, and 6.5 months of age administered with either AAV-EGFP (EGFP) or AAV-E2F4DN (E2F4DN) at 1.5 months, revealed with anti-Aβ, APP or Lamin B1 (loading control) antibodies. Asterisk: Aβ band (4 kDa). Arrow: processed Aβ monomers. (**b**, **c**) Ratio with Lamin B1 of Aβ monomer (4 kDa; b) or APP (**c**) in hippocampal extracts from h5xFAD mice administered with either AAV-EGFP (EGFP) or AAV-E2F4DN (E2F4DN) at the indicated ages. A significant correlation with time was observed for the level of Aβ monomer in both experimental situations. A statistically significant decrease of the regression slope was observed in E2F4DN-treated mice (*t* = 4.651209, *p* = 0.00164210; *t* test). N.S. = nonsignificant. (**d**) Aβ amyloid deposits in the hippocampus of 6.5-month-old h5xFAD mice administered with either vehicle or AAV-E2F4DN (hE2F4DN) at 1.5 months, stained with thioflavin S (arrows). DG = dentate gyrus; CA1 = cornu ammonis 1; Sub = subiculum. Scale bar, 50 μm. (**e**) Quantification of percentage of total area occupied by the Aβ deposits in the hippocampus. *p < 0.05 (two-tailed Student’s *t* test)

Neuronal expression of murine E2F4DN-myc in transgenic mice leads to slowdown of Aβ accumulation in 5xFAD mice at 3–6 months of age [19]. Consistently, we found that the intravenous administration of AAV-hE24DN in 1.5-month-old h5xFAD mice leads to a time-dependent reduction of Aβ production in the hippocampus (Fig. 3a, b). In contrast, APP expression was unaffected by age and the presence of E2F4DN in this tissue (Fig. 3a, c). As expected, the reduction of Aβ production in the presence of E2F4DN correlated with the decrease of Aβ accumulation in the hippocampus of 6.5-month-old mice as evidenced by thioflavin S staining (Fig. 3d, e), an accepted method for the identification of dense core plaques [55]. Therefore, the beneficial effects triggered by the expression of hE2F4DN in adult neurons include the stabilization of normal APP metabolism that decreases both Aβ production and accumulation, thus preventing this classical hallmark of AD.

### Systemic Delivery of hE2F4DN Using the AAV.PHP.B Vector Attenuates Microgliosis and Reactive Astrogliosis in h5xFAD Mice

**Fig. 4:**
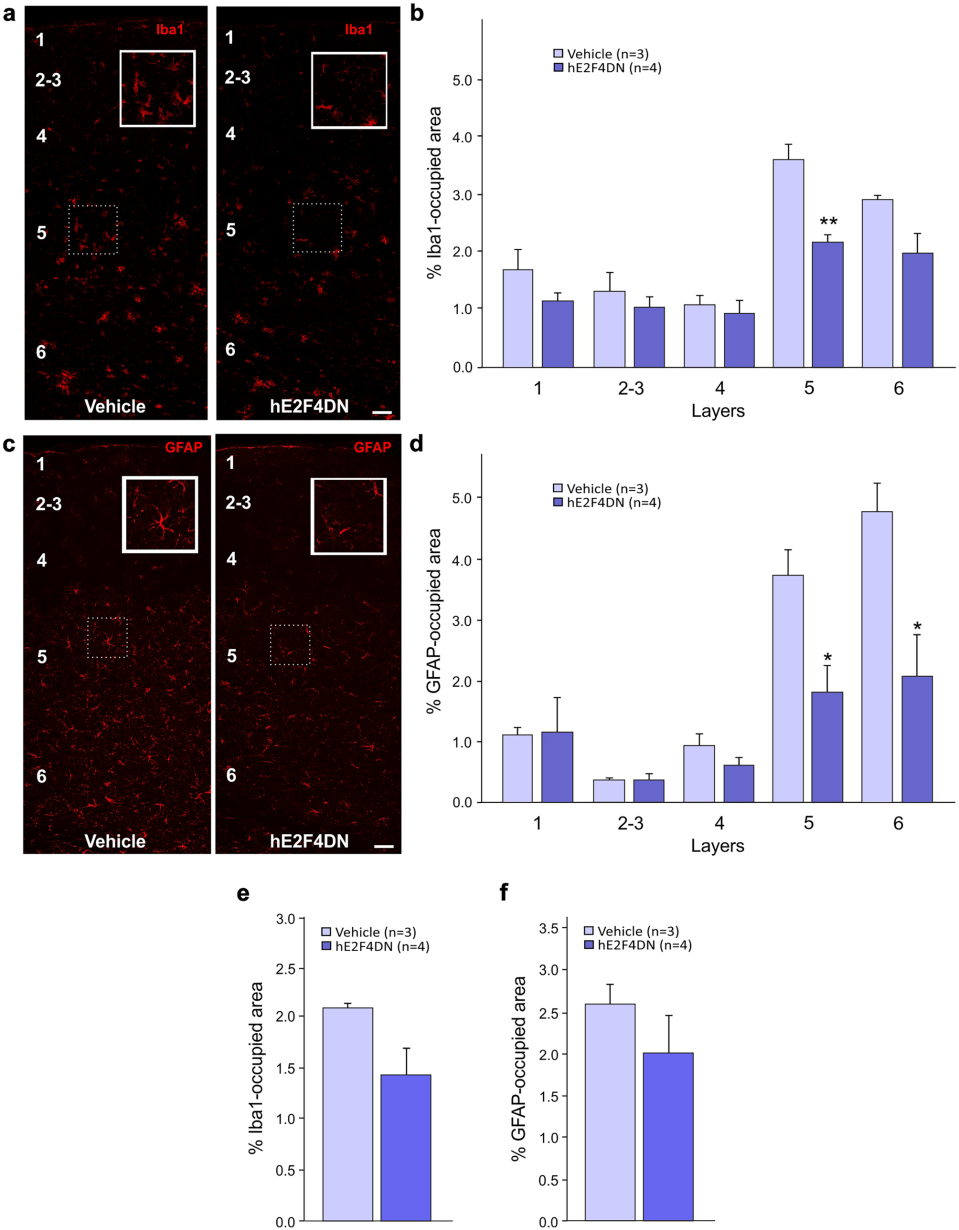
Systemic administration of the AAV-hE2F4DN vector attenuates microgliosis and reactive astrocytosis in the cerebral cortex of h5xFAD mice. (**a**) Representative Iba1 immunostaining in the cerebral cortex of 6.5-month-old h5xFAD mice administered with either vehicle or AAV-hE2F4DN at 1.5 months. Notice the decreased area occupied by Iba1-positive labeling in the cerebral cortex of h5xFAD mice expressing neuronal hE2F4DN. Inserts show Iba1 staining of the indicated dashed boxes. Layers are indicated with Arabic numbers. (**b**) Percentages of the areas occupied by Iba1 immunostaining in the indicated cortical layers (*F*_(1,25)_ = 20.1462; *p* = 0.0001; unbalanced two-way ANOVA). Numbers refer to the different cortical layers. (**c**) Representative GFAP immunostaining in the cerebral cortex of 6.5-month-old h5xFAD mice administered with either vehicle or AAV-hE2F4DN at 1.5 months. Notice the decreased GFAP-positive labeling and reactivity of astrocytes in the cerebral cortex of h5xFAD mice expressing neuronal hE2F4DN. Inserts show GFAP staining of the indicated dashed boxes. Layers are indicated with Arabic numbers. (**d**) Percentages of the areas occupied by GFAP immunostaining in the indicated cortical layers (*F*_(1,25)_ = 13.9501; *p* = 0.001; unbalanced two-way ANOVA). Numbers refer to the different cortical layers. *p < 0.05, **p < 0.01; unbalanced two-way ANOVA followed by post hoc two-tailed Student’s *t* test. Scale bar, 50 μm. (**e**, **f**) Systemic administration of the AAV-hE2F4DN vector leads to a tendency for the attenuation of microgliosis and reactive astrocytosis in the hippocampus of h5xFAD mice. Percentage of area occupied by Iba1 immunostaining (**e**). Percentage of area occupied by GFAP immunostaining (**f**)

Intravenous administration of AAV-hE24DN in 1.5-month-old h5xFAD mice reduced the area occupied by the microglia specific marker Iba1 in the cerebral cortex of 6.5-month-old h5xFAD mice as compared with control mice (Fig. 4a, b), an effect that was more pronounced in layer 5. Therefore, neuronal expression of hE2F4DN can attenuate microgliosis in the cerebral cortex of h5xFAD mice. A nonsignificant tendency for a reduced area occupied by Iba1 was also observed in the hippocampus of 6.5-month-old h5xFAD mice administered with AAV-hE24DN at 1.5 months (Fig. 4e).

Neuroinflammation is also accompanied by the presence of reactive astrocytes that, in the cerebral cortex, are detected as GFAP-positive cells [56]. We studied whether intravenous administration of AAV-hE24DN in 1.5-month-old h5xFAD mice can also reduce the area occupied by GFAP-positive immunostaining in the cerebral cortex of 6.5-month-old h5xFAD mice. As shown in Fig. 4c, d, the expression of hE2F4DN was able to significantly reduce the area occupied by GFAP, mostly in layers 5 and 6. We concluded that neuronal expression of hE2F4DN is able to attenuate reactive astrogliosis in the cerebral cortex of h5xFAD mice. Moreover, a nonsignificant tendency to a reduced area occupied by GFAP was also observed in the hippocampus of 6.5-month-old h5xFAD mice administered with AAV-hE24DN at 1.5 months (Fig. 4f).

### Systemic Delivery of hE2F4DN Using the AAV.PHP.B Vector Blocks Neuronal Tetraploidization in h5xFAD Mice

Cell cycle re-entry in neurons can lead to neuronal tetraploidization [57], a process that can be prevented by E2F4DN-myc in 5xFAD mice [19]. Therefore, we studied whether AAV-E2F4DN could also reverse this process in h5xFAD mice. To this aim, we used a flow cytometry-based method that has been validated by our laboratory [41, 42] and others [58, 59].

**Fig. 5:**
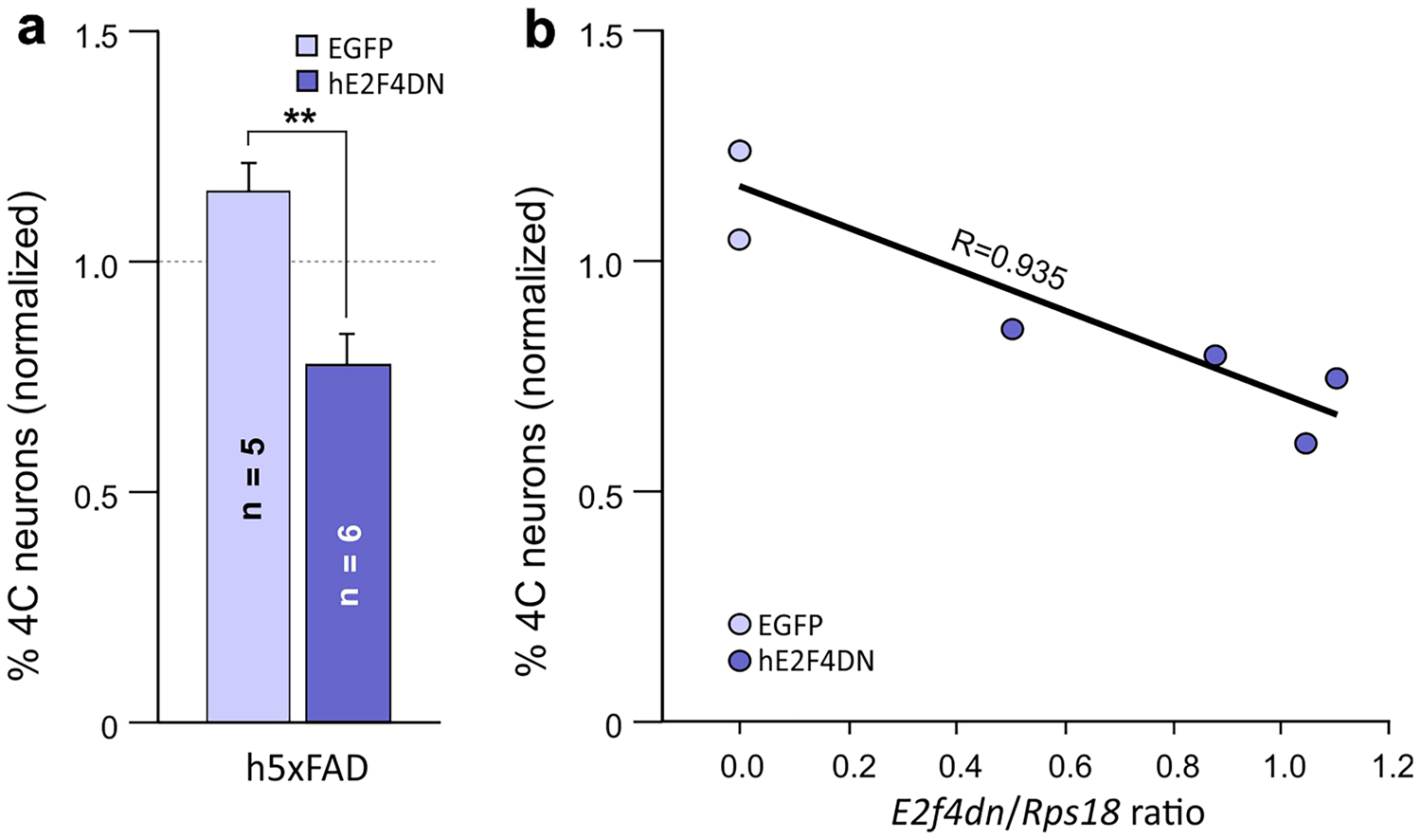
Systemic administration of the AAV-hE2F4DN vector prevents NT in h5xFAD mice. (**a**) NT quantification, normalized to NT levels in the cerebral cortex of 2-month-old WT mice, in cell nuclear extracts from the cerebral cortex of 2-month-old h5xFAD mice subjected to systemic administration of the AAV-EGFP control vector or the AAV-hE2F4DN vector at 1.5 months of age. Dashed line: NT in the cerebral cortex of 2-month-old WT mice. (*t*(9) = 4.66173, *p* = 0.0012; two-tailed Student’s *t* test). (**b**) Percentage of tetraploid neurons, normalized to NT levels in the cerebral cortex of 2-month-old WT mice, plotted against the expression levels of *E2f4dn* mRNA (normalized to *Rps10* rRNA). A statistically significant correlation was observed (*p* < 0.005; Pearson’s *R* correlation test)

The administration of AAV-hE2F4DN to 1.5-month-old h5xFAD mice significantly reduced the percentage of cortical tetraploid neurons found at 2 months of age when compared with the control situation (AAV-EGFP injection) (Fig. 5a). Therefore, human E2F4DN has an equivalent capacity to murine E2F4DN to block AD-associated NT in h5xFAD transgenic mice [19]. This conclusion is further supported by the statistically significant correlation observed between the proportion of tetraploid neurons and the *E2f4dn*/*Rps18* ratio in the cerebral cortex of 2-month-old h5xFAD mice injected at 1.5 months with AAV-E2FDN or AAV-EGFP (Fig. 5b).

### Systemic Delivery of hE2F4DN Using the AAV.PHP.B Vector Prevents Spatial Memory Deficits in h5xFAD Mice

**Fig. 6:**
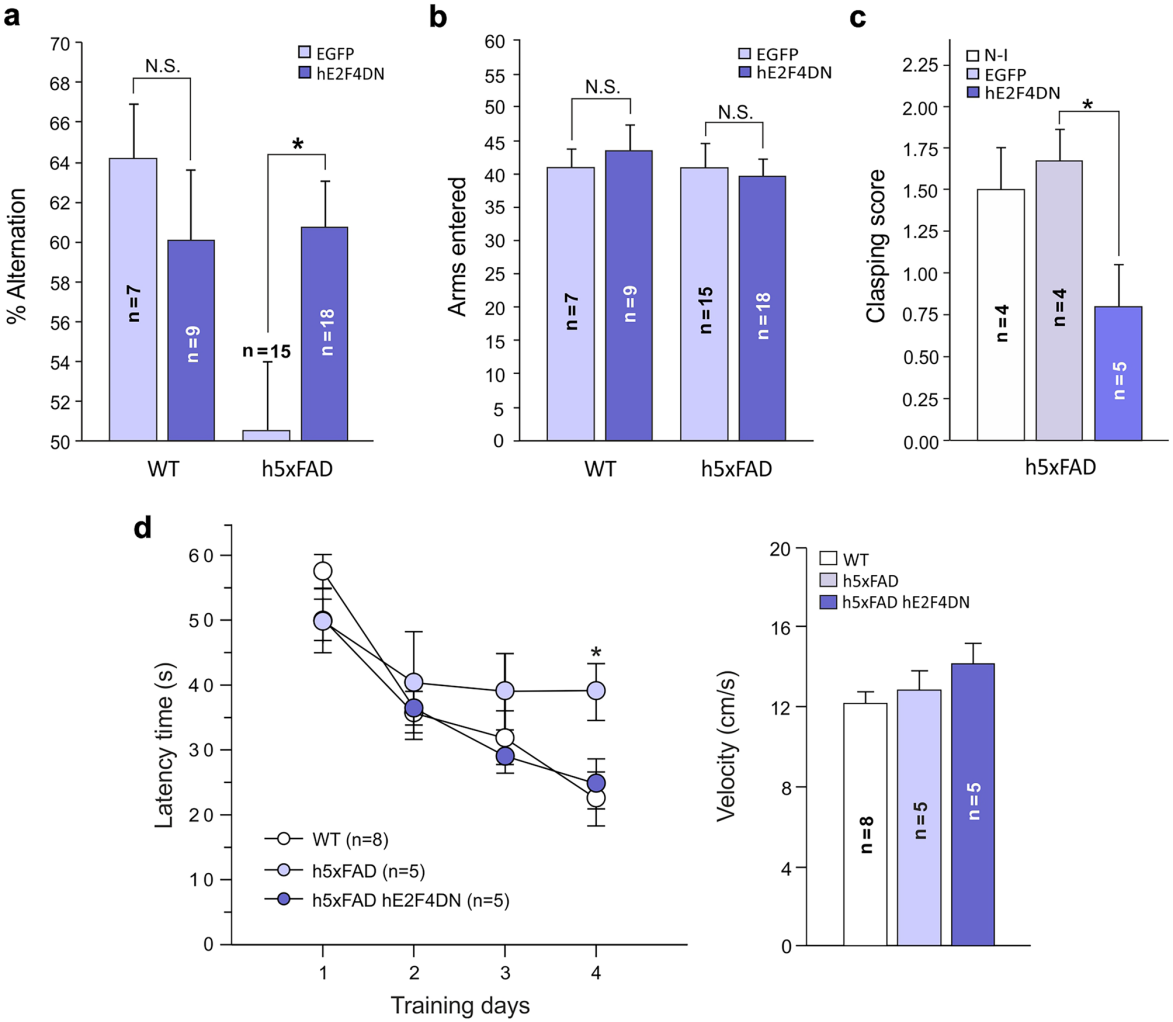
Systemic administration of the AAV-hE2F4DN vector prevents memory deficits and clasping behavior in h5xFAD mice. (**a**) Percentage of alternation in the Y-maze test of 3-month-old h5xFAD transgenic mice subjected to systemic administration of the AAV-EGFP control vector or the AAV-hE2F4DN vector. (*F*_(3,45)_ = 3.75619, *p* = 0.017; one-way ANOVA; WT/EGFP *vs* h5xFAD/EGFP: *q* = 13.742, *p* = 0.036; h5xFAD/EGFP *vs* h5xFAD/hE2F4DN: *q* = 10.204, *p* = 0.043; Tukey’s HSD post hoc test). (**b**) Number of arms entered in the Y-maze test of 3-month-old h5xFAD transgenic mice subjected to systemic administration of the AAV-EGFP control vector or the AAV-hE2F4DN vector. (*F*_(3,45)_ = 0.20931, *p* = 0.889; one-way ANOVA). (**c**) Analysis of paw-clasping behavior in h5xFAD mice of 9 months, either noninjected (N-I) or injected with AAV-EGFP (EGFP) or AAV-hE2F4DN (hE2F4DN). (h5xFAD/EGFP *vs* h5xFAD/hE2F4DN: K(9) = 4.267, *p* = 0.039; Kruskal–Wallis test). (**d**) hE2F4DN expression prevents spatial memory impairment in homozygous 5xFAD mice as evaluated by the MWM test at 6 months of age. Latency time to reach the hidden platform along four training days in homozygous 5xFAD mice either noninjected or injected with the AAV-hE2F4DN vector and in noninjected wild-type mice (left). Statistical significant differences between WT and h5xFAD mice, but not between WT and h5xFAD mice administered with AAV-E2F4DN, were observed at training day 4 (*F*_(2,15)_ = 4.0569, *p* = 0.039; one-way ANOVA; h5xFAD *vs* WT: *q* = 16.4475, *p* = 0.037; Tukey’s HSD post hoc test]. No statistically significant differences among experimental groups were observed for velocity of swimming (one-way ANOVA) (right). *p < 0.05 (Tukey’s HSD post hoc test)

To study whether neuronal expression of hE2F4DN can block the spatial memory impairment occurring in h5xFAD mice [35], 3-month-old h5xFAD and WT mice, to whom either AAV-hE2F4DN or AAV-EGFP vectors had been intravenously administered at 1.5 months of age, were subjected to the Y-maze test [44]. This test is based on the natural capacity of mice to remember the sequence of arms entry in a Y-shaped maze with three arms oriented in a 120° angle, commonly known as spontaneous alternations. This sequence is randomly chosen when the animals cannot remember, thus reducing the probability of alternation to 50%. This analysis confirmed that, at 3 months of age, h5xFAD mice show reduced probability of alternation under control conditions (AAV-EGFP injection) when compared with WT mice (Fig. 6a). In contrast, systemic administration of AAV-hE2F4DN fully reversed the spatial memory deficits of h5xFAD mice (Fig. 6a), an observation that was not dependent on the number of arms that were entered (Fig. 6b).

Systemic administration of AAV-hE2F4DN also improved training performance of h5xFAD mice in the MWM paradigm [43] carried out at 6 months of age. In this paradigm, learning is measured as the progressive reduction of swimming time required to reach a transparent platform where mice can escape from a circular water-filled pool containing visual labels. h5xFAD mice treated with AAV-hE2F4DN at 1.5 months showed a rapid decrease in the escape latency, similar to what was observed in WT mice (Fig. 6d, left). In contrast, noninjected h5xFAD mice showed severe learning deficit, which was significantly different at the fourth day of training when compared with WT mice (Fig. 6d, left). As a control, the velocity of swimming during the training period did not differ when the three experimental groups were compared with each other (Fig. 6d, right). Altogether, we conclude that both working memory and spatial learning loss are restored in h5xFAD administered with the AAV-hE2F4DN vector.

### Systemic Delivery of hE2F4DN Using the AAV.PHP.B Vector Prevents Paw-Clasping Behavior and Body Weight Loss in h5xFAD Mice

h5xFAD mice show a strong paw-clasping phenotype from 5 months onwards [34]. To verify whether the systemic administration of the AAV-hE2F4DN vector can prevent this phenotype, we performed a clasping test to 9-month-old h5xFAD mice previously subjected to systemic administration of either AAV-hE2F4DN or AAV-EGFP at 1.5 months or left noninjected. In parallel, 9-month-old WT mice were also subjected to the paw-clasping test, which scored 0 ± 0 (*n* = 6) as expected from the WT situation at this age. In contrast, both noninjected and control, AAV-EGFP-injected h5xFAD mice showed a strong paw-clasping phenotype, which was significantly reduced in h5xFAD mice injected with the AAV-hE2F4DN vector (Fig. 6c).

**Fig. 7:**
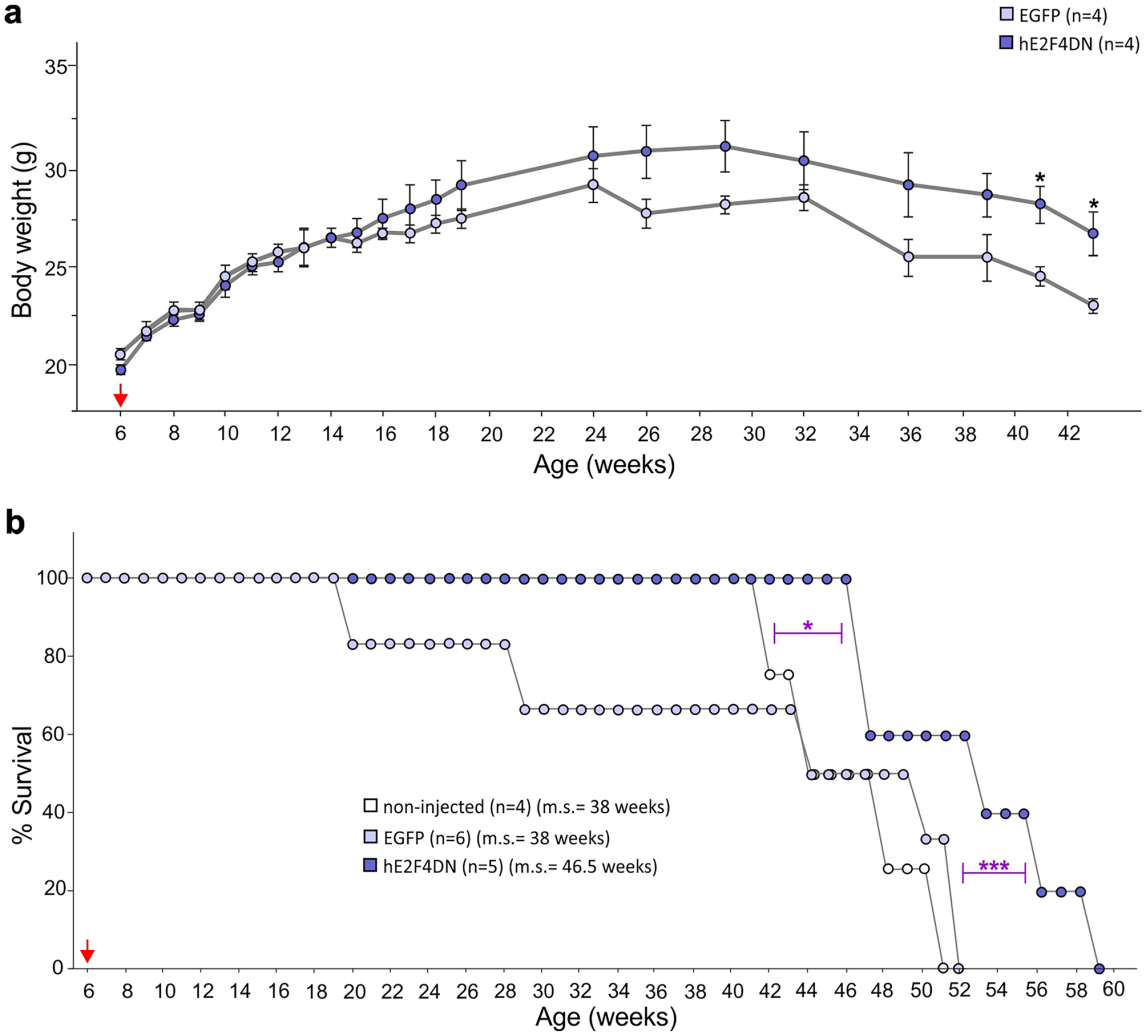
Systemic administration of the AAV-hE2F4DN vector prevents body weight loss and increases life expectancy in male h5xFAD mice. (**a**) Body weight at the indicated time points of 5xFAD mice (littermates) administered with either AAV-EGFP or AAV-hE2F4DN. (41 weeks: *t*(6) = 3.5032, *p* = 0.013; 43 weeks: *t*(6) = 3.1741, *p* = 0.019; two-tailed Student’s *t* test). (**b**) Survival expectancy in h5xFAD mice either noninjected or administered with AAV-EGFP or AAV-hE2F4DN vectors. (non-injected vs hE2F4DN: χ_(1)_ = 6.298; EGFP vs hE2F4DN: *χ*_(1)_ = 46.28; log-rank (Mantel-Cox) test). ***p < 0.001, *p < 0.05. Red arrow: time point at which the AAVs were administered. m.s. = median survival

The therapeutic effect of hE2F4DN in h5xFAD mice did not result in detectable adverse effects. Body weight gain in h5xFAD mice administered with the AAV-hE2F4DN vector was similar to control h5xFAD mice during the first 8 weeks after AAV administration (Fig. 7a). Nevertheless, h5xFAD mice injected with AAV-EGFP vector showed a tendency to lower body weight gain at ages older than 14 weeks, whereas they lost body weight from 32 weeks on, an effect that was mitigated by hE2F4DN (Fig. 7a). The lack of body weight gain observed in h5xFAD mice older than 24 weeks correlated with low life expectancy. Less than 50% of AAV-EGFP-treated mice were alive at 44 weeks, and none of them was alive by 52 weeks (Fig. 7b). Importantly, AAV-hE2F4DN administration statistically significantly extended life expectancy of h5xFAD mice. More than 50% of AAV-hE2F4DN-treated h5xFAD were alive at 52 weeks of age, and they were able to survive at least until 58 weeks (Fig. 7b). As a control, we also compared the survival of AAV-hE2F4DN-treated h5xFAD mice with noninjected h5xFAD mice since GFP transduction can cause cellular damage [54]. This analysis confirmed that the expression of hE2F4DN results in longer life expectancy (Fig. 7b) (respective median survivals in weeks for noninjected, EGFP and hE2F4DN treated h5xFAD mice, 38, 38, 46.5). Therefore, we conclude that the hE2F4DN-based therapy has beneficial effects not only in cognition but also in other h5xFAD-associated features such as abnormal reflexes (paw-clasping), body weight loss, and reduced life expectancy.

### Systemic Delivery of hE2F4DN Using the AAV.PHP.B Vector Does Not Affect Thr249 Phosphorylation of Endogenous E2F4 in h5xFAD Mice

A previous study from our laboratory provided evidence that E2F4 becomes Thr phosphorylated in AD mouse models [32]. This latter study, based on a proximity ligation assay with antibodies against E2F4 and phosphoThr, does not demonstrate that the observed Thr-specific phosphorylation occurs within the Thr249/Thr251 motif since mouse E2F4 contains other Thr residues susceptible to become phosphorylated [27]. To directly proof that E2F4 becomes phosphorylated in the Thr249/Thr251 motif, we raised an antibody specific for mouse phosphoThr249-E2F4 (see “Materials and Methods” section). Western blot analysis performed in hippocampal extracts from 4.5-month-old mice with this antibody revealed a main band with apparent molecular weight of ~150 kDa, which was also detected using an E2F4 specific antibody (Fig. S7a). This band likely corresponds to an E2F4 trimer, as a similar band could be detected when recombinant hE2F4DN-StrepII was subjected to 3–4 rounds of thawing/freezing (Fig. S7a). This suggests that E2F4 phosphorylation within the Thr249/Thr251 motif leads to a conformational change in E2F4 that favors its trimerization, a circumstance of unclear significance. This hypothetical conformational change would be mimicked by recombinant hE2F4DN-StrepII subjected to 3–4 freezing/thawing cycles. In addition to the ~150-kDa band observed in both WT and h5xFAD mice, the phosphoThr249-specific antibody also identified a ~50-kDa band specifically in hippocampal extracts from h5xFAD mice. This band, which shows higher mobility than recombinant hE2F4DN-strepII, can also be detected with an anti-E2F4 antibody in hippocampal extracts from both WT and h5xFAD mice (Fig. S7a). This suggests that a modified version of E2F4 becomes phosphorylated in the hippocampus of h5xFAD mice. This supports the hypothesis that the phosphorylation of E2F4 within its Thr249/Thr251 motif is enhanced in h5xFAD mice.

hE2F4DN lacks its Thr248/Thr250 motif but not its putative docking domain for p38^MAPK^ [31]. Therefore, hE2F4DN could sequester p38^MAPK^, thus preventing the phosphorylation of endogenous E2F4 by this kinase. To test whether the mechanism of action of hE2F4DN is based on the inhibition of endogenous E2F4 phosphorylation, we performed western blots using hippocampal extracts from 4.5-month-old 5xFAD mice that were either noninjected or administered with the AAV-hE2F4DN vector at 1.5 months of age. This analysis indicated that the expression of hE2F4DN, which showed an apparent molecular weight similar to that of recombinant hE2F4DN-StrepII, does not prevent the phosphorylation of endogenous E2F4 within its Thr conserved motif in both ~150 and ~50 kDa bands (Fig. S7b). We conclude that, when expressed in neurons, the unphosphorylated form of hE2F4 has potential to prevent AD-associated pathology without affecting the phosphorylation of endogenous E2F4. Therefore, our results suggest that E2F4 phosphorylation in AD favors a pathological phenotype, which is consistent with the phosphorylation observed in the cerebral cortex of h5xFAD mice (Fig. S7a), whereas the expression of dephosphorylated form of hE2F4 reverses this situation.

## Discussion

E2F4 is a transcription factor with a crucial role in the control of quiescence in vertebrate cells [60]. In the nervous system, E2F4 participates in neuronal differentiation [61], and its expression is maintained in adult neurons from nonhuman primates [62], where it could participate in protective and homeostatic processes [63, 64], being potentially linked to AD [20]. This makes this protein an attractive therapeutic target for complex neurodegenerative conditions such as AD.

E2F4 is a phosphoprotein with Thr248 (in human) and Thr249 (in mouse) being the major phosphorylatable Thr residue [27], and E2F4 phosphorylation can lead to cell cycle progression [65, 66]. We have previously shown that chicken E2F4 has two Thr residues (Thr261/Thr263) whose phosphorylation is relevant for NT since the E2F4 phosphomimetic containing Thr261Glu/Thr263Glu mutations induces cell cycle re-entry in differentiating chick neurons [31]. In addition, a chicken form of E2F4DN having its Thr261/Thr263 residues substituted by Ala can block NT [31]. These Thr residues are conserved in vertebrates [31], and previous results indicate that equivalent mutations in the conserved Thr motif of mouse E2F4 (Thr249/Thr251) mitigate multiple processes that are altered in AD when this murine variant is expressed in neurons from 5xFAD transgenic mice using the *Mapt* promoter [19, 33]. This suggests that the phosphorylation of these Thr residues may prevent the function of E2F4 as a homeostasis and quiescence keeper in neurons. We provide evidence for this hypothesis as the phosphorylation levels of endogenous E2F4 (50 kDa band) increase in the hippocampus of 5xFAD mice. We have also provided evidence that hE2F4DN expression cannot prevent the phosphorylation of the endogenous E2F4 protein. Therefore, the mechanism of action of our therapeutic agent could derive from its known capacity to regulate genes involved in AD-associated processes [19, 26] or its potential interaction with synaptic proteins [27]. Moreover, E2F4DN might antagonize the deleterious effect of E2F1 on cognition [42], as occurs in other paradigms in which E2F1 and E2F4 show opposite effects [60, 67]. Future work will test these hypotheses.

In this study, we have tested whether a human form of E2F4DN unable to become phosphorylated in Thr248/Thr250 could be employed as a therapeutic molecule. Our results demonstrate that the AAV-hE2F4DN vector efficiently crosses the BBB in mice, as previously shown by Deverman et al. [33] and specifically transduces a significant percentage of neurons, even in mature mice of 8.5 months of age. The avoidance of E2F4DN expression in glial cells or precursors involved in adult neurogenesis prevents undesirable side effects. Moreover, and contrary to the observation of Jackson et al. [51], who found an attenuation of long-term expression with the *hSyn1* promoter in rats, our results indicate that hE2F4 expression is effective already two weeks after administration and remains at high levels at least 1 year after administration, the latest time point studied. Therefore, one single administration of this vector can be used for long-lasting, generalized transgene expression in the brain [68]. Finally, despite that hE2F4DN-myc was observed to be expressed by a negligible proportion of hepatocytes or cardiomyocytes, no side effects were detected, and mice expressing E2F4DN-myc or E2F4DN lived longer and showed reduced body weight lost.

Forced cell cycle re-entry in neurons results in classical hallmarks of AD, including tau protein hyperphosphorylation and neurofibrillary tangles (NFT), increased APP processing and extracellular deposits of Aβ, neuronal cell death, gliosis, and cognitive deficits [69–72], and it has been shown to enhance the phenotype of a murine model of AD [73]. Musi et al. [74] have recently shown that genes involved in cell cycle progression are upregulated in human NFT-containing neurons. Moreover, a detailed proteomic analysis performed in six different brain regions from AD patients, as compared with control individuals, has identified several signaling pathways involved in cell cycle regulation as being widely dysregulated in severely affected regions of the AD brain [75]. Altogether, this evidence indicates that NT might participate in the etiology of AD.

We have found that NT precedes the neuropathology and the known deficits in spatial learning observed in both homozygous (this study) and hemizygous 5xFAD mice [19]. This suggests that NT could be associated with the cognitive deficits observed in AD, as previously shown for ageing-associated cognitive loss [42]. In this regard, we have shown that forced cell cycle re-entry in cultured cortical neurons correlates with synaptic dysfunction [14]. The reduced capacity of hyperploid neurons to fire action potentials could perturb the neuronal networks where they are integrated, a mechanism that may account for the cognitive loss observed in AD [14, 15]. Therefore, the improvement of the cognitive phenotype observed in E2F4DN-expressing h5xFAD mice could partially derive from the capacity of this molecule to block NT. Nevertheless, other effects of E2F4DN likely account for its beneficial effect on cognition. For instance, we have shown that the expression of hE2F4DN leads to reduced expression and accumulation of Aβ in the hippocampus, a finding consistent with the control by E2F4DN of gene networks involved in processing, accumulation and toxicity of Aβ, which resulted in the attenuation of Aβ deposition into dense core plaques in hemizygous 5xFAD mice of 6 months of age [19].

As previously observed in E2F4FDN knock-in mice [19], neuroinflammation was attenuated in h5xFAD mice treated with AAV.E2F4DN. A statistically significant decrease was observed in the area occupied by both Iba1- and GFAP-specific immunoreactivity in the cerebral cortex of h5xFAD mice administered with AAV.E2F4DN. This indicates that microgliosis and reactive astrogliosis are both mitigated by neuronal expression of hE2F4DN, likely through well-established mechanisms of bidirectional neuron-glia communication [76].

Our results demonstrate that the therapeutic approach based on neuronal hE2F4DN expression using the AAV.PHP.B vector correlates with prevention of the spatial learning deficits observed in h5xFAD mice, as demonstrated with cognitive tasks evaluating working memory (spontaneous alternation Y-maze test) and reference memory (MWM test). This observation confirms those reported by López-Sánchez et al. [19], who demonstrated that neuronal expression of murine E2F4DN-myc improves cognition in hemizygous 5xFAD mice as evidenced by the Y-maze paradigm. In addition, neuronal E2F4DN expression also reverses the paw-clasping and low-anxiety behaviors observed in h5xFAD mice [34].

Our gene therapeutic approach based on hE2F4DN expression in neurons was also able to reduce the weight loss phenotype observed in 5xFAD mice [45], which we have found to be exacerbated in h5xFAD mice. This observation confirms what was observed in 5xFAD/E2F4DN transgenic mice [19], thus indicating that our E2F4DN-based gene therapy might prevent metabolic alterations associated with AD [19].

Our results indicate that E2F4DN-based gene therapy represents a promising approach against AD, a condition susceptible to be targeted with AAV-based gene therapy [77], as other neurological disorders [78, 79]. Indeed, AAV-based gene therapy has already been approved for several clinical trials aimed at brain delivering of proteins. They include glutamic acid decarboxylase [80], aromatic L-amino acid decarboxylase [81], nerve growth factor [82], N-sulfoglycosamine sulfohydrolase/sulfatase-modifying factor 1 [83], neurturin [84], ApoE2 [85], and neuropeptide Y [86]. Nevertheless, the AAV.PHP.B vector can only cross the BBB in C57BL6 mice [87] and other mouse strains that express Ly6a [88, 89]. Accordingly, the AAV.PHP.B vector does not cross the BBB appreciably better than AAV9 in nonhuman primates [90, 91] (primates do not have Ly6a [92]) and thus will not translate to humans. Therefore, neuronal expression of hE2F4DN using AAV vectors with improved capacity to cross the BBB in humans is required to be used as a therapeutic approach for AD in the future.

Our treatment is performed in h5xFAD mice of 1.5 months, a presymptomatic stage in which intraneuronal Aβ accumulation is already observed and just 2 weeks before extracellular deposits of Aβ are increased 3.5 times as compared with heterozygous 5xFAD mice [34]. Therefore, if effective, this therapy could be applied to prodromal stages of AD. At present, it is unclear whether the administration of this therapy is able to reverse AD pathology in symptomatic mice.

In sum, our study has defined a novel approach against AD using a strategy with a multifactorial effect that is effective in preventing AD-associated alterations including cognitive impairment and body weight loss. We believe our approach will be useful for the treatment of this devastating neurodegenerative disease.

## Supplementary Information

Below is the link to the electronic supplementary material.

Supplementary file1 (DOCX 2629 KB)

Supplementary file2 (PDF 477 KB)

Supplementary file3 (PDF 493 KB)

Supplementary file4 (PDF 476 KB)

Supplementary file5 (PDF 476 KB)

Supplementary file6 (PDF 476 KB)

## References

[1] Brookmeyer R, Gray S, Kawas C (1998). Projections of Alzheimer’s disease in the United States and the public health impact of delaying disease onset. Am J Public Health.

[2] Förstl H, Kurz A (1999). Clinical features of Alzheimer’s disease. Eur. Arch. Psychiatry Clin Neurosci.

[3] Gallardo G, Holtzman DM (2019). Amyloid-β and Tau at the Crossroads of Alzheimer’s Disease. Adv Exp Med Biol.

[4] Tamura BK, Masaki KH, Blanchette P (2007). Weight loss in patients with Alzheimer’s disease. J Nutr Elder.

[5] Sergi G, De Rui M, Coin A, Inelmen EM, Manzato E (2013). Weight loss and Alzheimer’s disease: temporal and aetiologic connections. Proc Nutr Soc.

[6] Gong C-X, Liu F, Iqbal K (2018). Multifactorial hypothesis and multi-targets for Alzheimer’s disease. J Alzheimers Dis.

[7] Clare R, King VG, Wirenfeldt M, Vinters HV (2010). Synapse loss in dementias. J Neurosci Res.

[8] Duran-Aniotz C, Hetz C (2016). Glucose Metabolism: A sweet relief of Alzheimer’s disease. Curr Biol.

[9] Tönnies E, Trushina E (2017). Oxidative stress, synaptic dysfunction, and Alzheimer’s disease. J Alzheimers Dis.

[10] Thal DR, Capetillo-Zarate E, Larionov S, Staufenbiel M, Zurbruegg S, Beckmann N (2009). Capillary cerebral amyloid angiopathy is associated with vessel occlusion and cerebral blood flow disturbances. Neurobiol Aging.

[11] Sala Frigerio C, Wolfs L, Fattorelli N (2019). The major risk factors for Alzheimer’s disease: age, sex, and genes modulate the microglia response to Aβ plaques. Cell Rep.

[12] Li P, Marshall L, Oh G (2019). Epigenetic dysregulation of enhancers in neurons is associated with Alzheimer’s disease pathology and cognitive symptoms. Nat Commun.

[13] Frade JM, López-Sánchez N (2017). Neuronal tetraploidy in Alzheimer and aging. Aging.

[14] Barrio-Alonso E, Hernández-Vivanco A, Walton CC, Perea G, Frade JM (2018). Hyperploidy triggers synaptic dysfunction and delayed cell death in differentiated cortical neurons. Sci Rep.

[15] Barrio-Alonso E, Fontana B, Valero M, Frade JM (2020). Pathological aspects of neuronal hyperploidization in Alzheimer’s disease evidenced by computer simulation. Front Genet.

[16] Yang Y, Geldmacher DS, Herrup K (2001). DNA replication precedes neuronal cell death in Alzheimer’s disease. J Neurosci.

[17] Arendt T, Brückner MK, Mosch B, Lösche A (2010). Selective cell death of hyperploid neurons in Alzheimer’s disease. Am J Pathol.

[18] Cacabelos R, Alvarez A, Fenández-Novoa L, Lombardi VR (2000). A pharmacogenomic approach to Alzheimer’s disease. Acta Neurol Scand Suppl..

[19] López-Sánchez N, Ramón-Landreau M, Trujillo C, Garrido-García A, Frade JM. A single multifactorial target against Alzheimer’s Disease. bioRxiv 2020;2020.05.08.082784. 10.1101/2020.05.08.082784.

[20] Ding J, Kong W, Mou X, Wang S (2019). Construction of transcriptional regulatory network of Alzheimer’s disease based on PANDA Algorithm Interdiscip Sci.

[21] Kong W, Mou X, Zhi X, Zhang X, Yang Y. Dynamic regulatory network reconstruction for Alzheimer’s disease based on matrix decomposition techniques. Comput Math Methods Med 2014;2014:891761. 10.1155/2014/891761 PMC4082865 25024739

[22] Orr AL, Kim C, Jimenez-Morales D (2019). Neuronal Apolipoprotein E4 Expression Results in Proteome-Wide Alterations and Compromises Bioenergetic Capacity by Disrupting Mitochondrial Function. J. Alzheimers Dis.

[23] Caldwell AB, Liu Q, Schroth GP, et al. Dedifferentiation and neuronal repression define familial Alzheimer’s disease. Sci Adv 2020;6:eaba5933. 10.1126/sciadv.aba5933 PMC7673760 33188013

[24] Augustin R, Lichtenthaler SF, Greeff M, Hansen J, Wurst W, Trümbach D. Bioinformatics identification of modules of transcription factor binding sites in Alzheimer’s disease-related genes by in silico promoter analysis and microarrays. Int J Alzheimers Dis 2011;2011:154325. 10.4061/2011/154325 PMC3090009 21559189

[25] Karch CM, Ezerskiy LA, Bertelsen S Alzheimer’s Disease Genetics Consortium (ADGC), A. M. Goate, Alzheimer’s disease risk polymorphisms regulate gene expression in the ZCWPW1 and the CELF1 loci. PLoS One 2016;11:e0148717. 10.1371/journal.pone.0148717 PMC4769299 26919393

[26] Lee BK, Bhinge AA, Iyer VR (2011). Wide-ranging functions of E2F4 in transcriptional activation and repression revealed by genome-wide analysis. Nucleic Acids Res.

[27] Hsu J, Arand J, Chaikovsky A (2019). E2F4 regulates transcriptional activation in mouse embryonic stem cells independently of the RB family. Nat Commun.

[28] Chen X, Ma W, Zhang S, Paluch J, Guo W, Dickman DK. The BLOC-1 subunit pallidin facilitates activity-dependent synaptic vesicle recycling. eNeuro 2017;4:ENEURO.0335–16.2017. 10.1523/ENEURO.0335-16.2017 PMC5356223 28317021

[29] Desrosiers RR, Fanélus I (2011). Damaged proteins bearing L-isoaspartyl residues and aging: a dynamic equilibrium between generation of isomerized forms and repair by PIMT. Curr Aging Sci.

[30] Sahlan M, Zako T, Yohda M (2018). Prefoldin, a jellyfish-like molecular chaperone: functional cooperation with a group II chaperonin and beyond. Biophys Rev.

[31] Morillo SM, Abanto EP, Román MJ, Frade JM (2012). Nerve growth factor-induced cell cycle reentry in newborn neurons is triggered by p38^MAPK^-dependent E2F4 phosphorylation. Mol Cell Biol.

[32] López-Sánchez N, Frade JM. [P2–139]: A mutant form of E2F4 prevents neuronal tetraploidization and cognitive deficits in 5xFAD mice without affecting Aβ deposition. Alzheimers Dement 2017;13 (7S Part 13):P659-P661. Abstract. 10.1016/j.jalz.2017.06.789

[33] Deverman BE, Pravdo PL, Simpson BP (2016). Cre-dependent selection yields AAV variants for widespread gene transfer to the adult brain. Nat Biotechnol.

[34] Richard BC, Kurdakova A, Baches S, Bayer TA, Weggen S, Wirths O (2015). Gene Dosage Dependent Aggravation of the Neurological Phenotype in the 5XFAD Mouse Model of Alzheimer’s Disease. J. Alzheimers Dis.

[35] Oakley H, Cole SL, Logan S (2006). Intraneuronal beta-amyloid aggregates, neurodegeneration, and neuron loss in transgenic mice with five familial Alzheimer’s disease mutations: potential factors in amyloid plaque formation. J Neurosci.

[36] Czernik AJ, Mathers J, Mische SM. Phosphorylation state-specific antibodies. In: *Neuromethods: Regulatory Protein Modification*. Techniques & Protocols 1997;30:219–250.

[37] Gibson DG, Young L, Chuang RY, Venter JC, Hutchison CA3rd, Smith HO. Enzymatic assembly of DNA molecules up to several hundred kilobases. Nat Methods 2009;6:343–345. 10.1038/nmeth.1318 19363495

[38] Kügler S, Meyn L, Holzmüller H (2001). Neuron-specific expression of therapeutic proteins: evaluation of different cellular promoters in recombinant adenoviral vectors. Mol Cell Neurosci.

[39] Schambach A, Bohne J, Baum C (2006). Woodchuck hepatitis virus post-transcriptional regulatory element deleted from X protein and promoter sequences enhances retroviral vector titer and expression. Gene Ther.

[40] Schambach A, Galla M, Maetzig T, Loew R, Baum C (2007). Improving transcriptional termination of self-inactivating gamma-retroviral and lentiviral vectors. Mol Ther.

[41] López-Sánchez N, Frade JM (2013). Genetic evidence for p75^NTR^-dependent tetraploidy in cortical projection neurons from adult mice. J Neurosci.

[42] López-Sánchez N, Fontán-Lozano Á, Pallé A (2017). Neuronal tetraploidization in the cerebral cortex correlates with reduced cognition in mice and precedes and recapitulates Alzheimer’s-associated neuropathology. Neurobiol Aging.

[43] Morris RG, Garrud P, Rawlins JN, O’Keefe J (1982). Place navigation impaired in rats with hippocampal lesions. Nature.

[44] Miller RC, Miller EK (1970). Lack of a long-term effect of LSD on Y-maze learning in mice. Nature.

[45] Jawhar S, Trawicka A, Jenneckens C, Bayer TA, Wirths O (2012). Motor deficits, neuron loss, and reduced anxiety coinciding with axonal degeneration and intraneuronal Aβ aggregation in the 5XFAD mouse model of Alzheimer’s disease. Neurobiol Aging.

[46] Donello JE, Loeb JE, Hope TJ (1998). Woodchuck hepatitis virus contains a tripartite posttranscriptional regulatory element. J Virol.

[47] Loeb JE, Cordier WS, Harris ME, Weitzman MD, Hope TJ (1999). Enhanced expression of transgenes from adeno-associated virus vectors with the woodchuck hepatitis virus posttranscriptional regulatory element: implications for gene therapy. Hum Gene Ther.

[48] Choi JH, Yu NK, Baek GC (2014). Optimization of AAV expression cassettes to improve packaging capacity and transgene expression in neurons. Mol Brain.

[49] Glover CP, Bienemann AS, Heywood DJ, Cosgrave AS, Uney JB (2002). Adenoviral-mediated, high-level, cell-specific transgene expression: a SYN1-WPRE cassette mediates increased transgene expression with no loss of neuron specificity. Mol Ther.

[50] Rincon MY, de Vin F, Duqué SI, et al. Widespread transduction of astrocytes and neurons in the mouse central nervous system after systemic delivery of a self-complementary AAV-PHP.B vector. Gene Ther 2018;25:83–92. 10.1038/s41434-018-0005-z 29523880

[51] Jackson KL, Dayton RD, Deverman BE, Klein RL. Better targeting, better efficiency for wide-scale neuronal transduction with the synapsin promoter and AAV-PHP.B. Front Mol Neurosci 2016;9:116. 10.3389/fnmol.2016.00116 PMC5095393 27867348

[52] Lalonde R, Strazielle C (2011). Brain regions and genes affecting limb-clasping responses. Brain Res Rev.

[53] Walf AA, Frye CA (2007). The use of the elevated plus maze as an assay of anxiety-related behavior in rodents. Nat Protoc.

[54] Ansari AM, Ahmed AK, Matsangos AE (2016). Cellular GFP Toxicity and Immunogenicity: Potential Confounders in in Vivo Cell Tracking Experiments. Stem Cell Rev Rep.

[55] McLellan ME, Kajdasz ST, Hyman BT, Bacskai BJ (2003). In vivo imaging of reactive oxygen species specifically associated with thioflavine S-positive amyloid plaques by multiphoton microscopy. J Neurosci.

[56] Miyake T, Hattori T, Fukuda M, Kitamura T, Fujita S (1988). Quantitative studies on proliferative changes of reactive astrocytes in mouse cerebral cortex. Brain Res.

[57] Frade JM, Ovejero-Benito MC (2015). Neuronal cell cycle: the neuron itself and its circumstances. Cell Cycle.

[58] Sigl-Glöckner J, Brecht M (2017). Polyploidy and the Cellular and Areal Diversity of Rat Cortical Layer 5 Pyramidal Neurons. Cell Rep.

[59] Nandakumar S, Grushko O, Buttitta LA. Polyploidy in the adult Drosophila brain. Elife 2020;9:e54385. 10.7554/eLife.54385 PMC7447450 32840209

[60] Miles S, Breeden L (2017). A common strategy for initiating the transition from proliferation to quiescence. Curr Genet.

[61] Persengiev SP, Kondova II, Kilpatrick DL (1999). E2F4 actively promotes the initiation and maintenance of nerve growth factor-induced cell differentiation. Mol Cell Biol.

[62] Morgan KL, Chalovich EM, Strachan GD, Otis LL, Jordan-Sciutto KL (2005). E2F4 expression patterns in SIV encephalitis. Neurosci Lett.

[63] Liu DX, Nath N, Chellappan SP, Greene LA (2005). Regulation of neuron survival and death by p130 and associated chromatin modifiers. Genes Dev.

[64] Hsu J, Sage J (2016). Novel functions for the transcription factor E2F4 in development and disease. Cell Cycle.

[65] Garneau H, Paquin MC, Carrier JC, Rivard N (2009). E2F4 expression is required for cell cycle progression of normal intestinal crypt cells and colorectal cancer cells. J Cell Physiol.

[66] Paquin MC, Cagnol S, Carrier JC, Leblanc C, Rivard N (2013). ERK-associated changes in E2F4 phosphorylation, localization and transcriptional activity during mitogenic stimulation in human intestinal epithelial crypt cells. BMC Cell Biol.

[67] Paramio JM, Segrelles C, Casanova ML, Jorcano JL (2000). Opposite functions for E2F1 and E2F4 in human epidermal keratinocyte differentiation. J Biol Chem.

[68] Lim JA, Yi H, Gao F, Raben N, Kishnani PS, Sun B. Intravenous injection of an AAV-PHP.B vector encoding human acid α-glucosidase rescues both muscle and CNS defects in murine Pompe disease. Mol Ther Methods Clin Dev 2019;12:233–245. 10.1016/j.omtm.2019.01.006 PMC6376130 30809555

[69] Lee HG, Casadesus G, Nunomura A (2009). The neuronal expression of Myc causes a neurodegenerative phenotype in a novel transgenic mouse. Am J Pathol.

[70] McShea A, Lee HG, Petersen RB (2007). Neuronal cell cycle re-entry mediates Alzheimer disease-type changes. Biochim Biophys Acta.

[71] Park KH, Hallows JL, Chakrabarty P, Davies P, Vincent I (2007). Conditional neuronal simian virus 40 T antigen expression induces Alzheimer-like tau and amyloid pathology in mice. J Neurosci.

[72] Park KHJ, Barrett T (2020). Gliosis Precedes Amyloid-β Deposition and Pathological Tau Accumulation in the Neuronal Cell Cycle Re-Entry Mouse Model of Alzheimer’s Disease. J Alzheimers Dis Rep.

[73] Barrett T, Stangis KA, Saito T, Saido T, Park KHJ. Neuronal cell cycle re-entry enhances neuropathological features in AppNLF knock-in mice. J Alzheimers Dis 2021;82:1683-1702. 10.3233/JAD-210091 PMC8461670 34219712

[74] Musi N, Valentine JM, Sickora KR, et al. Tau protein aggregation is associated with cellular senescence in the brain. Aging Cell 2018;17:e12840. 10.1111/acel.12840 PMC6260915 30126037

[75] Xu J, Patassini S, Rustogi N (2019). Regional protein expression in human Alzheimer’s brain correlates with disease severity. Commun Biol.

[76] Simon E, Obst J, Gomez-Nicola D (2019). The evolving dialogue of microglia and neurons in Alzheimer’s disease: microglia as necessary transducers of pathology. Neuroscience.

[77] Loera-Valencia R, Piras A, Ismail MAM (2018). Targeting Alzheimer’s Disease with Gene and Cell Therapies. J Intern Med.

[78] Hudry E, Vandenberghe LH (2019). Therapeutic AAV Gene Transfer to the Nervous System: A Clinical Reality. Neuron.

[79] Deverman BE, Ravina BM, Bankiewicz KS, Paul SM, Sah DWY (2018). Gene therapy for neurological disorders: progress and prospects. Nat Rev Drug Discov.

[80] Kaplitt MG, Feigin A, Tang C (2007). Safety and tolerability of gene therapy with an adeno-associated virus (AAV) borne GAD gene for Parkinson’s disease: an open label, phase I trial. Lancet.

[81] Muramatsu S, Fujimoto K, Kato S (2010). A phase I study of aromatic L-amino acid decarboxylase gene therapy for Parkinson’s disease. Mol Ther.

[82] Rafii MS, Baumann TL, Bakay RA (2014). A phase1 study of stereotactic gene delivery of AAV2-NGF for Alzheimer’s disease. Alzheimers Dement.

[83] Tardieu M, Zérah M, Husson B, et al. Intracerebral administration of adeno-associated viral vector serotype rh.10 carrying human SGSH and SUMF1 cDNAs in children with mucopolysaccharidosis type IIIA disease: results of a phase I/II trial. Hum Gene Ther 2014;25:506–516. 10.1089/hum.2013.238 24524415

[84] Marks WJJr, Baumann TL, Bartus RT. Long-Term Safety of Patients with Parkinson’s Disease Receiving rAAV2-Neurturin (CERE-120) Gene Transfer. Hum Gene Ther 2016;27:522–527. 10.1089/hum.2015.134 26711317

[85] Rosenberg JB, Kaplitt MG, De BP, et al. AAVrh.10-Mediated APOE2 Central Nervous System Gene Therapy for APOE4-Associated Alzheimer’s Disease. Hum. Gene Ther Clin Dev 2018;29:24–47. 10.1089/humc.2017.231 PMC5870071 29409358

[86] Patrício MI, Barnard AR, Green AL, During MJ, Sen A, MacLaren RE (2018). A clinical-grade gene therapy vector for pharmacoresistant epilepsy successfully overexpresses NPY in a human neuronal cell line. Seizure.

[87] Hordeaux J, Wang Q, Katz N, Buza EL, Bell P, Wilson JM. The Neurotropic Properties of AAV-PHP.B Are Limited to C57BL/6J Mice. Mol Ther 2018;26:664–668. 10.1016/j.ymthe.2018.01.018 PMC5911151 29428298

[88] Huang Q, Chan KY, Tobey IG, et al. Delivering genes across the blood-brain barrier: LY6A, a novel cellular receptor for AAV-PHP.B capsids. PLoS One 2019;14:e0225206. 10.1371/journal.pone.0225206 PMC6855452 31725765

[89] Batista AR, King OD, Reardon CP, et al. Ly6a Differential Expression in Blood-Brain Barrier Is Responsible for Strain Specific Central Nervous System Transduction Profile of AAV-PHP.B. Hum Gene Ther 2020;31:90–102. 10.1089/hum.2019.186 31696742

[90] Matsuzaki Y, Konno A, Mochizuki R, et al. Intravenous administration of the adeno-associated virus-PHP.B capsid fails to upregulate transduction efficiency in the marmoset brain. Neurosci Lett 2018;665:182–188. 10.1016/j.neulet.2017.11.049 29175632

[91] Liguore WA, Domire JS, Button D, Wang Y, Dufour BD, Srinivasan S, McBride JL. AAV-PHP.B Administration Results in a Differential Pattern of CNS Biodistribution in Non-human Primates Compared with Mice. Mol Ther 2019;27:2018–2037. 10.1016/j.ymthe.2019.07.017 PMC6838922 31420242

[92] Loughner CL, Bruford EA, McAndrews MS, Delp EE, Swamynathan S, Swamynathan SK (2016). Organization, evolution and functions of the human and mouse Ly6/uPAR family genes. Hum Genomics.

