# Supplementary material for: E2F4-Based Gene Therapy Mitigates the Phenotype of the Alzheimer’s Disease Mouse Model 5xFAD
Source: Neurotherapeutics. 2021 Nov 11;18(4):2484–503. doi: 10.1007/s13311-021-01151-1 (PMC8804140; doi:10.1007/s13311-021-01151-1)
Supplement: Supplementary file 1 — Supplementary file1 (DOCX 2629 KB) [file 13311_2021_1151_MOESM1_ESM.docx]

**SUPPLEMENTARY INFORMATION**

**E2F4-based gene therapy mitigates the phenotype of the Alzheimer’s disease mouse model 5xFAD**

Noelia López-Sánchez^1^, Alberto Garrido-García^1^, Morgan Ramón-Landreau^1^, Vanesa Cano-Daganzo^1^, José M. Frade^1^

^1^Department of Molecular, Cellular and Developmental Neurobiology, Cajal Institute, E-28002 Madrid, Spain

**Table S1.** Mice subjected to AAV.PHP.B-based delivery of E2F4DN-myc, EGFP, or E2F4DN.

| **Genotype** | **Treatment** | **Dose (vg/kg)** | **n** |
| --- | --- | --- | --- |
| Wild type | EGFP | 6.25x10^13^ | 10 |
| Wild type | E2F4DN-myc | 3.12x10^13^ | 7 |
| Wild type | E2F4DN-myc | 6.25x10^13^ | 35 |
| Wild type | E2F4DN-myc | 1.25x10^14^ | 7 |
| Wild type | E2F4DN | 6.25x10^13^ | 13 |
| h5xFAD | EGFP | 6.25x10^13^ | 21 |
| h5xFAD | E2F4DN | 6.25x10^13^ | 35 |

**Table S2.** Myc-positive cells from the indicated tissues after evaluating the specified number of sections from nine WT mice injected with AAV.PHP.B-E2F4DN-myc (6.25x10^13^ vg/kg) at 1.5 months of age and analyzed the indicated months post injection (mpi). Adr. Gl. (adrenal gland), Intest. (small intestine).

| **Tissue** | **1 mpi** | | | **3 mpi** | | | **6 mpi** | | |
| --- | --- | --- | --- | --- | --- | --- | --- | --- | --- |
|  | *Mouse#* | *Number of sections* | *Myc+ cells* | *Mouse#* | *Number of sections* | *Myc+ cells* | *Mouse#* | *Number of sections* | *Myc+ cells* |
| Heart | 1 | 29 | 0 | 5 | 15 | 0 | 8 | 16 | 1 |
|  | 2 | 30 | 0 | 6 | 12 | 0 | 9 | 16 | 0 |
|  | 3 | 27 | 0 | 7 | 15 | 0 | --- | --- | --- |
|  | 4 | 24 | 0 | --- | --- | --- | --- | --- | --- |
| Spleen | 1 | 24 | 0 | 5 | 9 | 0 | 8 | 18 | 0 |
|  | 2 | 24 | 0 | 6 | 9 | 0 | 9 | 12 | 0 |
|  | 3 | 24 | 0 | 7 | 12 | 0 | --- | --- | --- |
|  | 4 | 36 | 0 | --- | --- | --- | --- | --- | --- |
| Ovary | 1 | 24 | 0 | 5 | 24 | 0 | 8 | 12 | 0 |
|  | 2 | 28 | 0 | 6 | 30 | 0 | 9 | 18 | 0 |
|  | 3 | 26 | 0 | 7 | 21 | 0 | --- | --- | --- |
|  | 4 | 26 | 0 | --- | --- | --- | --- | --- | --- |
| Kidney | 1 | 24 | 0 | 5 | 12 | 0 | 8 | 24 | 0 |
|  | 2 | 24 | 0 | 6 | 12 | 0 | 9 | 18 | 0 |
|  | 3 | 24 | 0 | 7 | 11 | 0 | --- | --- | --- |
|  | 4 | 24 | 0 | --- | --- | --- | --- | --- | --- |
| Adr. Gl. | 1 | 22 | 0 | 5 | 12 | 0 | 8 | 15 | 0 |
|  | 2 | 24 | 0 | 6 | 12 | 0 | 9 | 16 | 0 |
|  | 3 | 24 | 0 | 7 | 11 | 0 | --- | --- | --- |
|  | 4 | 24 | 0 | --- | --- | --- | --- | --- | --- |
| Thymus | --- | --- | --- | --- | --- | --- | 8 | 24 | 0 |
|  | --- | --- | --- | --- | --- | --- | 9 | 24 | 0 |
| Muscle | --- | --- | --- | --- | --- | --- | 8 | 24 | 0 |
|  | --- | --- | --- | --- | --- | --- | 9 | 24 | 0 |
| Intest. | --- | --- | --- | --- | --- | --- | 8 | 22 | 0 |
|  | --- | --- | --- | --- | --- | --- | 9 | 24 | 0 |
| Lung | --- | --- | --- | --- | --- | --- | 8 | 18 | 0 |
|  | --- | --- | --- | --- | --- | --- | 9 | 24 | 0 |

**Table S3.** Number of Myc-positive hepatocytes per 10^5^ total hepatocytes at the indicated doses of the AAV-E2F4DN-myc vector. mpi: months post injection.

| **1 mpi** | | | **3 mpi** | **6 mpi** |
| --- | --- | --- | --- | --- |
| 3.12x10^13^ vg/kg | 6.25x10^13^ vg/kg | 12.5x10^13^ vg/kg | 6.25x10^13^ vg/kg | 6.25x10^13^ vg/kg |
| (n=4) | (n=4) | (n=4) | (n=3) | (n=2) |
| 14.34 ± 4.89 | 22.10 ± 3.14 | 46.66 ± 8.37 | 8.04 ± 1.57 | 8.31 ± 5.35 |


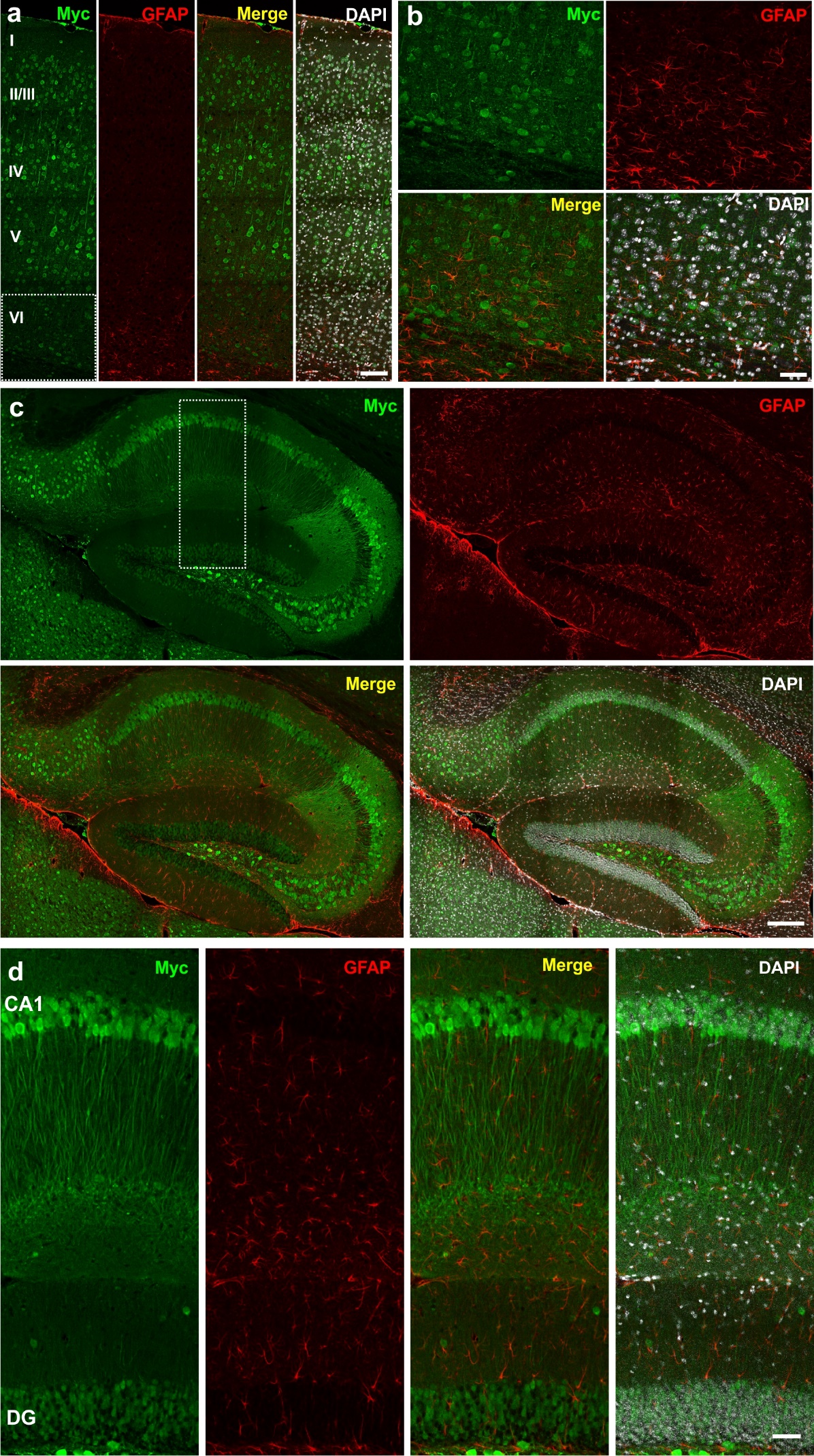


**Fig. S1.** E2F4DN-myc is not expressed by GFAP-positive cells in the cerebral cortex and hippocampus of WT mice subjected to systemic administration of the AAV-hE2F4DN-myc vector. Vibratome sections of the cerebral cortex (a,b) or hippocampus (c) from WT mice injected at 6 weeks of age with the AAV-hE2F4DN-myc vector (6.25x10^13^ vg/kg) and sacrificed 1 month later, immunostained with anti- anti-Myc tag (green) or GFAP (red). Notice that (b) illustrates the box shown in (a) and (d) the box shown in (c) . DAPI staining is shown in white.


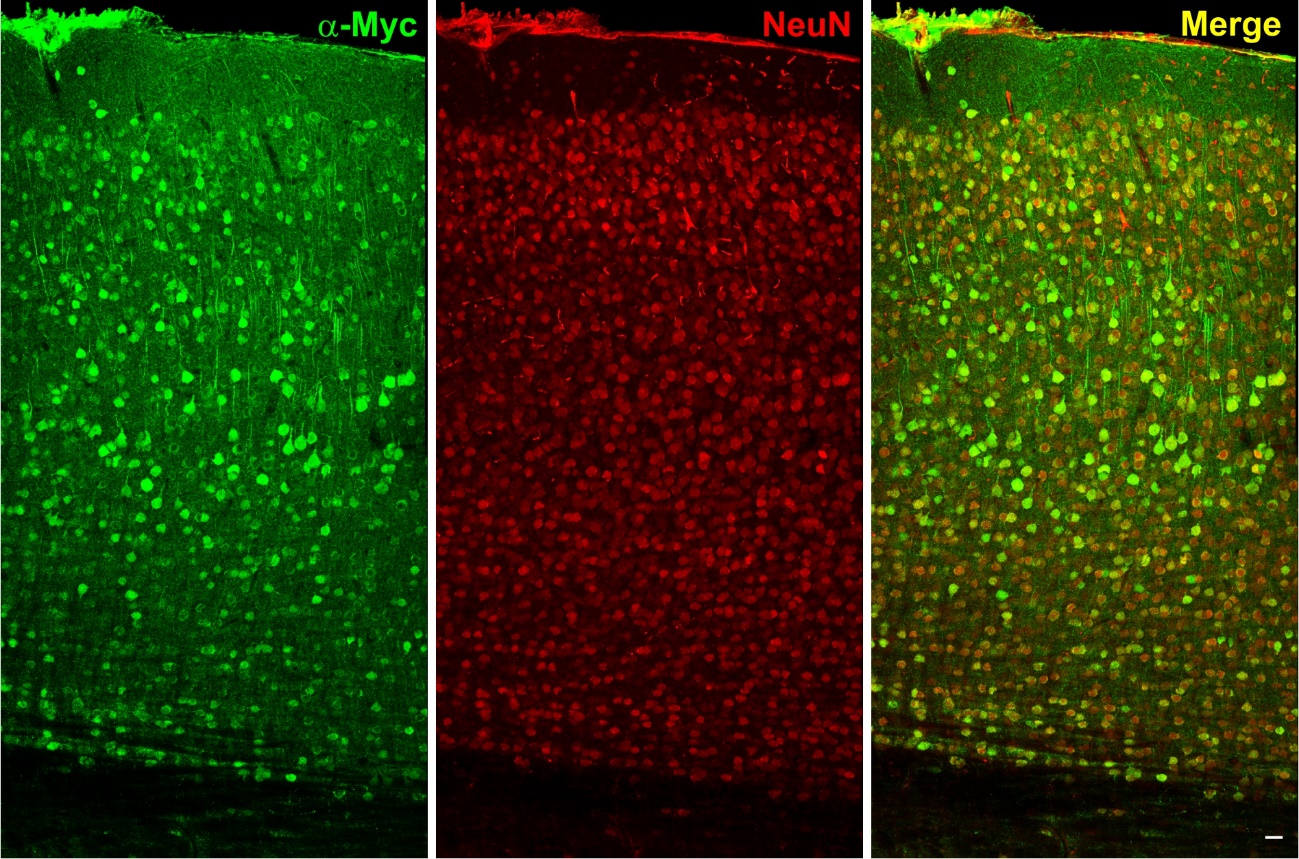


**Fig. S2.** Expression of E2F4DN-myc in the cerebral cortex of WT mice subjected to systemic administration of the AAV-hE2F4DN-myc vector. A vibratome section of the cerebral cortex from WT mice injected at 8.5 months of age with the AAV-hE2F4DN-myc vector (6.25x10^13^ vg/kg) and sacrificed 1 month later, immunostained with anti- anti-Myc tag (green) and NeuN (red).


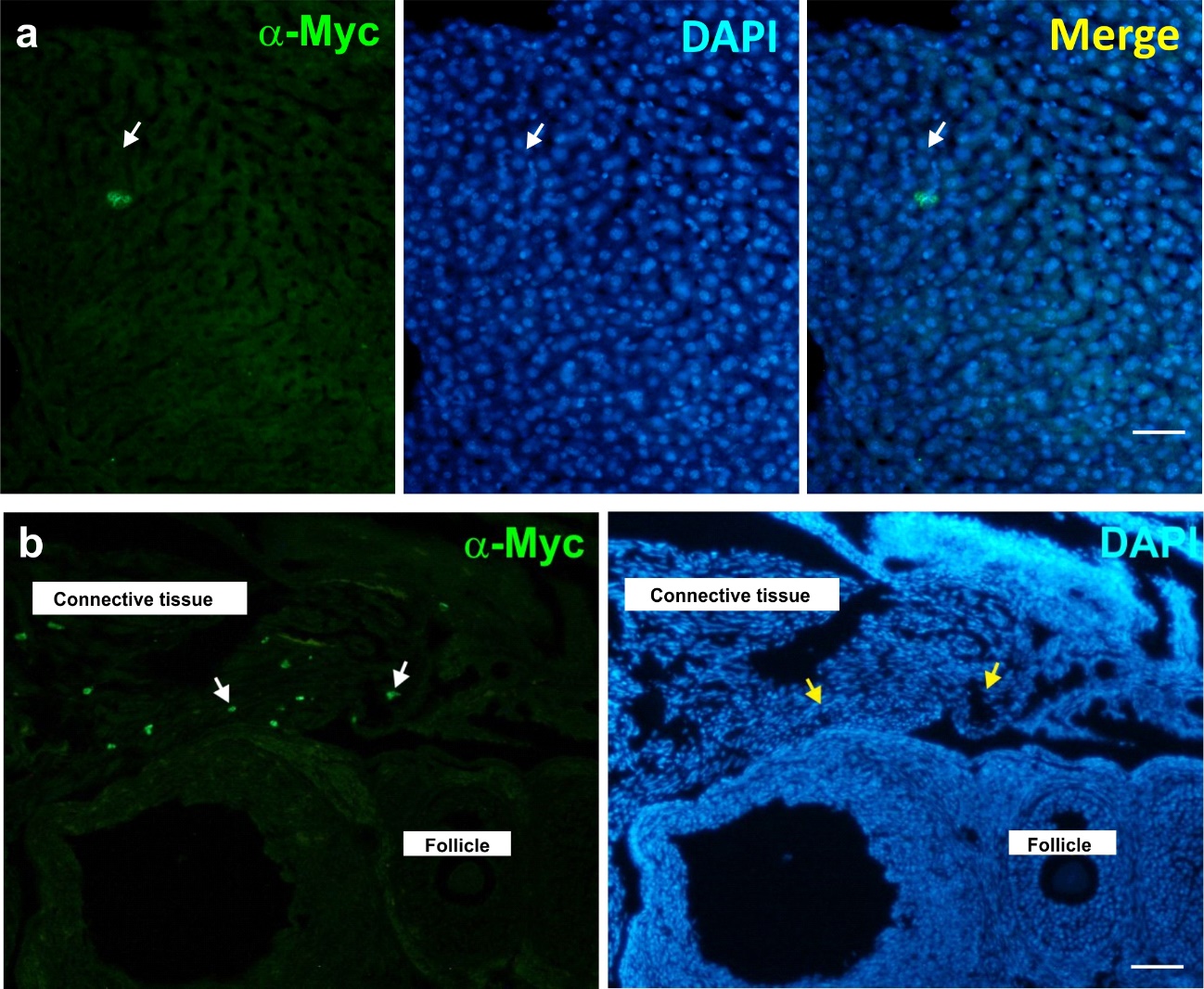


**Fig. S3** Expression of E2F4DN-myc in the liver of WT mice subjected to systemic administration of the AAV-E2F4DN-myc vector at 1.5 months of age. (a) Liver cryosections from WT mice sacrificed 3 months after systemic administration of the AAV-E2F4DN-myc vector immunostained with anti- anti-Myc tag (green). (b) Ovary cryosections from WT mice sacrificed 1 month after systemic administration of the AAV-E2F4DN-myc vector immunostained with anti- anti-Myc tag (green). Scale bar: 35 μm.


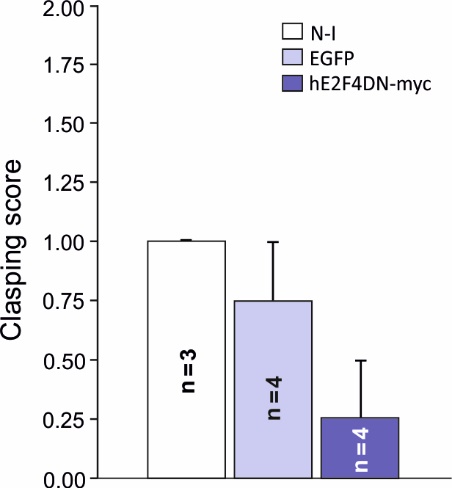


**Fig. S4** Analysis of paw-clasping behavior in 16 month-old WT mice, either non-injected (N-I) or injected with AAV-EGFP (EGFP) or AAV-hE2F4DN-myc (E2F4DN). Non-significant differences were observed among experimental groups (Kluskal-Wallis test).

**
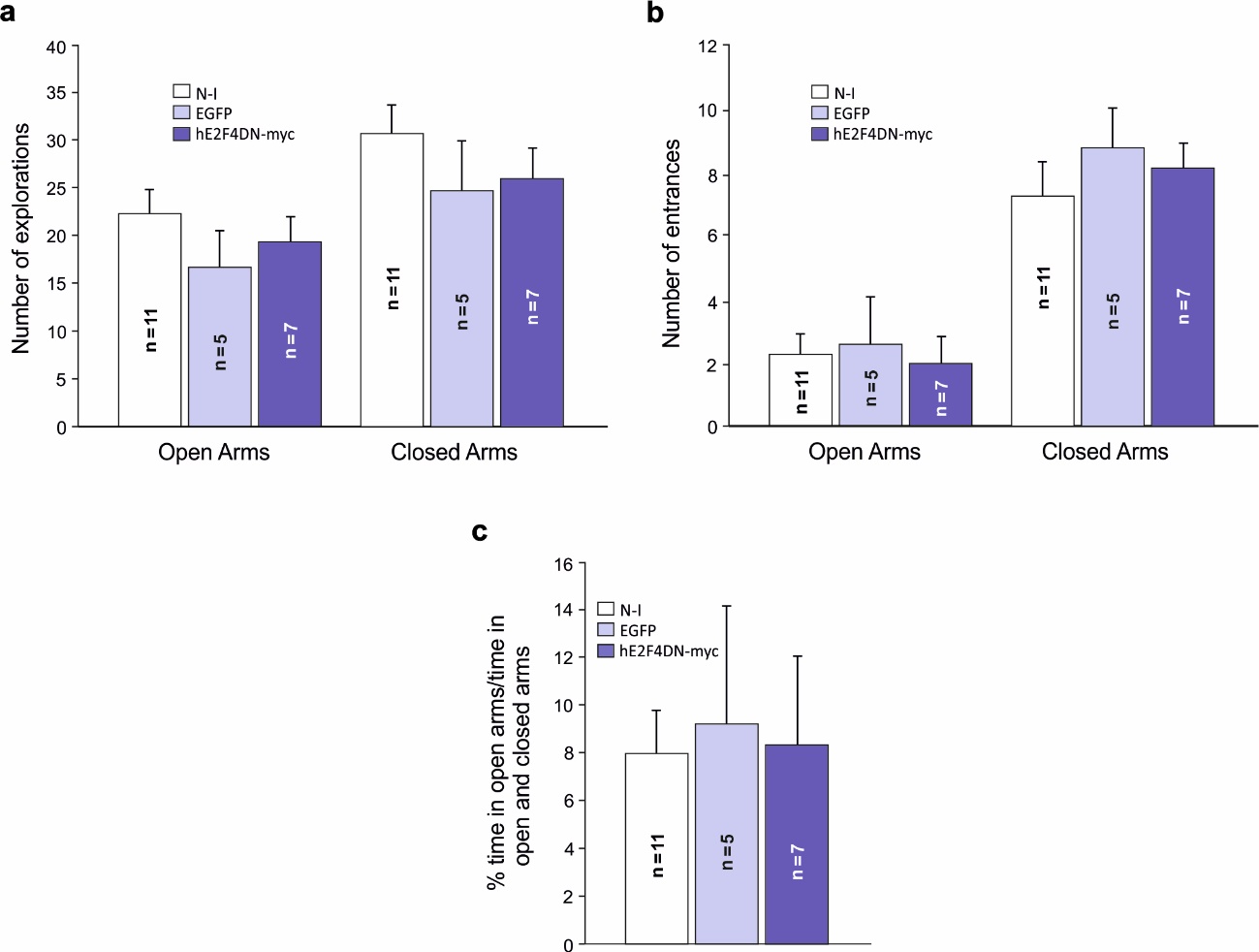
**

**Fig. S5** Anxiety analysis as measured by the EPM test in 12 month-old WT mice, either non-injected (N-I) or injected with AAV-EGFP (EGFP) or AAV-hE2F4DN-myc (E2F4DN-myc). No statistically significant differences among experimental groups were observed for the number of explorations (a), the number of entrances (b), or the percentage of time spent in the open arms relative to the time spent in the open and closed arms (c) [one-way ANOVA (a, closed arms in b), Kluskal-Wallis test (open arms in b, c)].

**
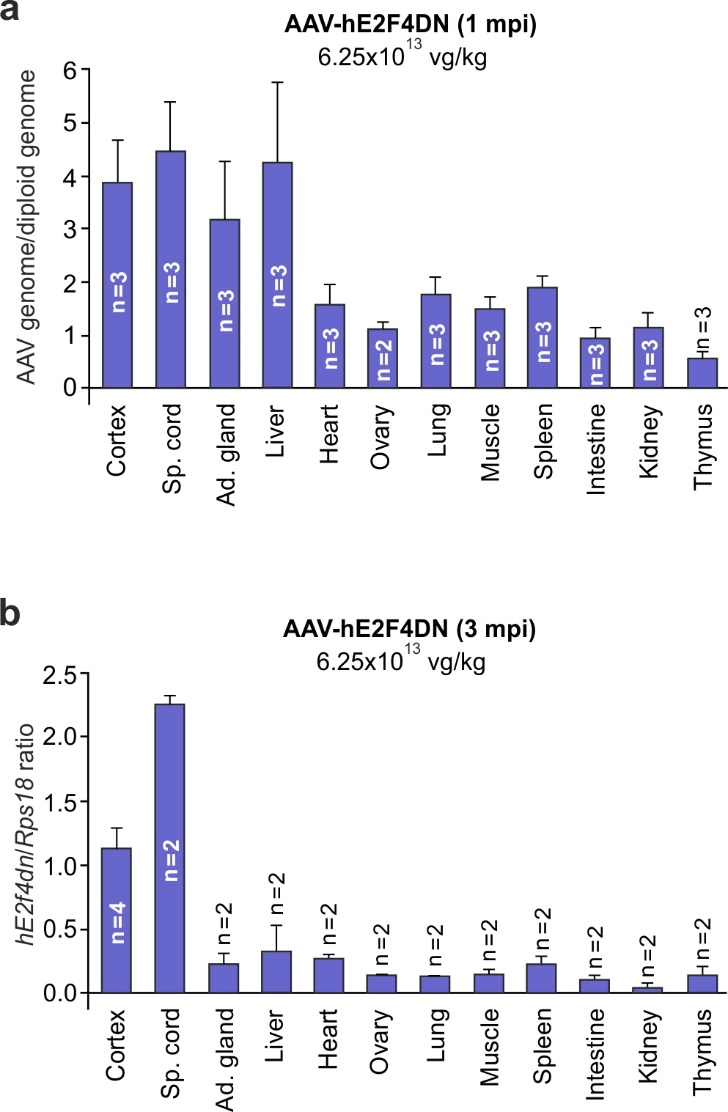
**

**Fig. S6** Biodistribution of AAV-E2F4DN genomic DNA and *hE2f4dn* mRNA. (a) qPCR analysis of AAV-E2F4DN genomic DNA in the indicated tissues 1 month post injection (mpi). qPCR values were normalized to an endogenous gene (*Kcn3a*) and, then, multiplied by 2 to estimate the levels per diploid genome. (b) qRT-PCR analysis of *hE2f4dn* mRNA in the indicated tissues 3 months post injection (mpi). qRT-PCR values were normalized to a house keeping gene mRNA levels (*Rps18*).

**
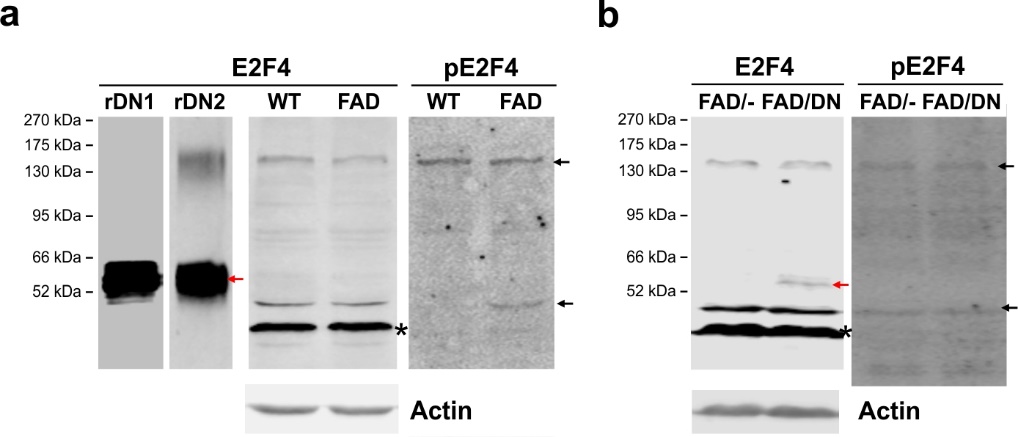
**

**Fig. S7** E2F4DN expression does not affect the phosphorylation stage of endogenous E2F4. (a) Representative western blots of recombinant E2F4-StrepII, non-manipulated (rDN1) or subjected to 3-4 freezing/thawing cycles (rDN2), and hippocampal extracts from either WT or 5xFAD (FAD) mice of 4.5 months of age, performed with anti-E2F4 (E2F4), anti-phosphoThr249-E2F4 (pE2F4), or anti-Actin antibodies. (b) Representative western blots of hippocampal extracts from 5xFAD mice of 4.5 months of age left untreated (FAD/-) or administered with the AAV-E2F4DN vector (FAD/DN), performed with anti-E2F4 (E2F4), anti-phosphoThr249-E2F4 (pE2F4), or anti-Actin antibodies. Black arrows: Thr249-phosphorylated E2F4; red arrow: exogenous E2F4DN (similar size as rDN1); *unspecific band.
